# Bitopic fluorescent antagonists of the A_2A_ adenosine receptor based on pyrazolo[4,3-*e*][1,2,4]triazolo[1,5-*c*]pyrimidin-5-amine functionalized congeners†
†Electronic supplementary information (ESI) available: Chemical synthesis, characterization data, HPLC analysis, pharmacological studies, off-target screening and additional molecular modeling procedures/results. 3D coordinates of the hA_2A_AR in complex with **11** (docking pose). 3D coordinates of the hA_2A_AR in complex with **12** (docking pose, BM2). Video of 30 ns of MD simulations of the hA_2A_AR in complex with **3**. Video S1. Trajectory visualization (left panel) and ligand–protein interaction energy profile (right panel) of 30 ns of membrane MD simulation of the **3**–hA_2A_AR complex. Video of 30 ns of MD simulations of hA_2A_AR in complex with **12**. Video S2. Trajectory visualization of 30 ns of membrane MD simulation of the **12**–hA_2A_AR complex starting from the docking binding mode denoted “BM2”: side view facing TM5, TM6, and TM7 (left panel), and side view facing TM4, TM5 and TM6 (right panel). See DOI: 10.1039/c7md00247e


**DOI:** 10.1039/c7md00247e

**Published:** 2017-06-22

**Authors:** Romain Duroux, Antonella Ciancetta, Philip Mannes, Jinha Yu, Shireesha Boyapati, Elizabeth Gizewski, Said Yous, Francisco Ciruela, John A. Auchampach, Zhan-Guo Gao, Kenneth A. Jacobson

**Affiliations:** a Molecular Recognition Section , Laboratory of Bioorganic Chemistry , National Institute of Diabetes and Digestive and Kidney Diseases , National Institutes of Health , Bldg. 8A, Rm. B1A-19 , Bethesda , MD 20892-0810 , USA . Email: kennethj@niddk.nih.gov ; Fax: +301 480 8422 ; Tel: +301 496 9024; b Inserm , CHU Lille , UMR-S 1172 - JPArc - Centre de Recherche Jean-Pierre AUBERT Neurosciences et Cancer , Univ. Lille , F-59000 Lille , France; c Department of Pharmaceutical Chemistry , Telangana University , Nizamabad , Telangana , India 503322; d Department of Pharmacology , Medical College of Wisconsin , 8701 Watertown Plank Road , Milwaukee , Wisconsin 53226 , USA; e Unitat de Farmacologia, Departament Patologia i Terapeutica Experimental , Facultat de Medicina , IDIBELL , Universitat de Barcelona , 08907 L'Hospitalet de Llobregat , Spain

## Abstract

A pyrazolo[4,3-*e*][1,2,4]triazolo[1,5-*c*]pyrimidin-5-amine antagonist of the A_2A_ adenosine receptor (AR) was functionalized as amine congeners, fluorescent conjugates and a sulfonate, and the A_2A_AR binding modes were predicted computationally. The optimal *n*-butyl spacer was incorporated into the following A_2A_AR-selective (*K*_i_, nM) conjugates: BODIPY630/650 derivative **11** (MRS7396, 24.6) and AlexaFluor488 derivative **12** (MRS7416, 30.3). Flow cytometry of **12** in hA_2A_AR-expressing HEK-293 cells displayed saturable binding (low nonspecific) and inhibition by known A_2A_AR antagonists. Water-soluble sulfonate **13** was a highly potent (*K*_i_ = 6.2 nM) and selective A_2A_AR antagonist based on binding and functional assays. Docking and molecular dynamics simulations predicted the regions of interaction of the distal portions of these chain-extended ligands with the A_2A_AR. The BODIPY630/650 fluorophore of **11** was buried in a hydrophobic interhelical (TM1/TM7) region, while AlexaFluor488 of **12** associated with the hydrophilic extracellular loops. In conclusion, we have identified novel high affinity antagonist probes for A_2A_AR drug discovery and characterization.

## Abstract 2

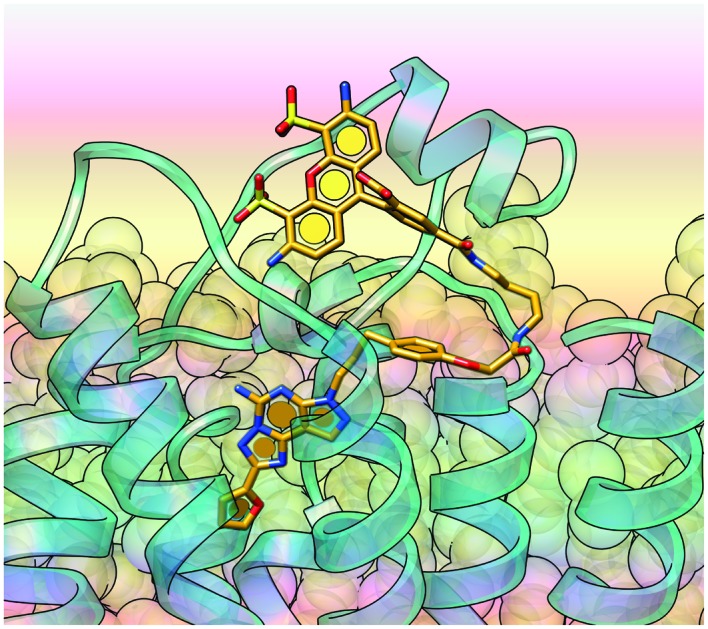
Functionalized antagonist probes of the A_2A_ adenosine receptor.

## Introduction

**Chart 1:**
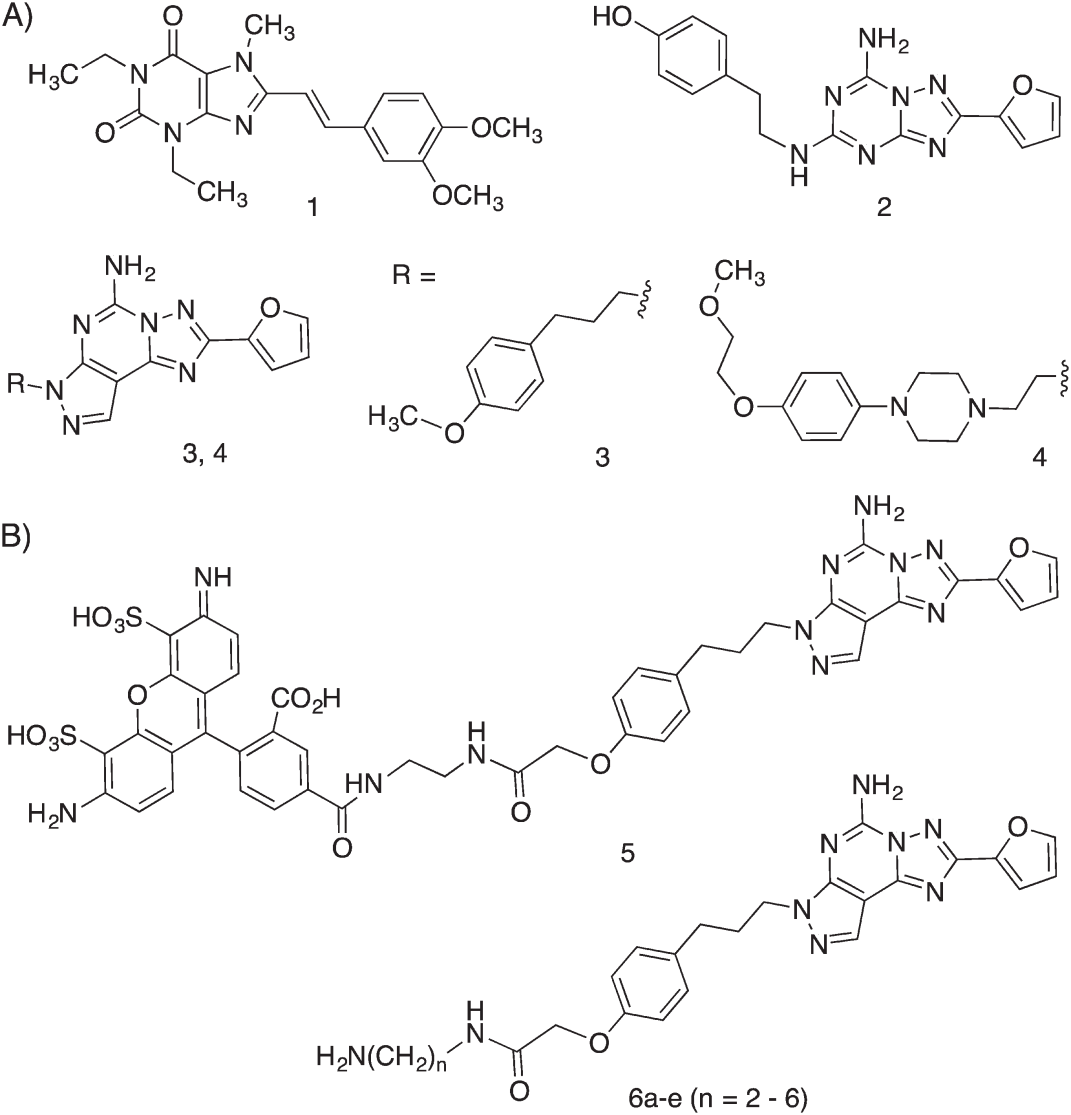
Structures of selective A_2A_AR antagonists and the target series: (A) widely used pharmacological probes and clinical candidates (**1–4**); (B) reported fluorescent probe **5** derived from **3** and functionalized amine congeners of varied chain length **6** explored in this study.

The development of selective agonists and antagonists of the four subtypes of adenosine receptors (ARs) has been extensively explored.^1^–^3^ Antagonists of the G_s_ protein-coupled A_2A_AR are sought for as agents for treating neurodegenerative conditions such as Parkinson's disease (PD) and Alzheimer's disease (AD), and for coadministration with cancer immunotherapy.^4^–^7^ Caffeine, the most consumed psychostimulant in the world, acts as a nonselective AR antagonist and readily enters the brain to antagonize the A_2A_AR at doses generally consumed. Epidemiological evidence showing a lower occurrence of AD and PD with modest caffeine intake points to the possibility that caffeine consumption is neuroprotective. Indeed, A_2A_AR antagonists can exert a neuroprotective effect on excitotoxicity in animal models; one mechanism appears to enhance the activity of an A_1_AR, which forms a heterodimer with the A_2A_AR, to inhibit glutamate release.^8^ A_2A_R antagonists also control microglia-mediated neuroinflammation.^9^ Therefore, it is possible that A_2A_AR antagonists may also delay neurodegenerative disease progression. In the striatum, A_2A_AR antagonists act to boost dopaminergic signaling, and thus provide symptomatic relief to reduce the motor deficits in PD without inducing dyskinesia. Certain selective A_2A_AR antagonists, *e.g.***1–4** (Chart 1), have been shown to enter the brain at sufficient levels for imaging and efficacy in PD models.^10^–^12^ A caffeine-like 1,3,7-trialkylxanthine, istradefylline **1**, is approved in Japan for treating PD, and its safety and efficacy in reducing in off-time (when PD symptoms return) in levodopa-treated patients were established in a 52 week trial.^10^ A tricyclic pyrazolo-triazolopyrimidine derivative **4**, which binds potently and selectively to the A_2A_AR, was in clinical trials for PD.^12^

In the periphery, elevated adenosine levels present in the microenvironment of tumors lead to a suppressed immune response, to shift the T cell response away from an aggressive state capable of attacking tumors.^6^ Increasing evidence supports the use of AR antagonists in cancer treatment, either with selectivity for the A_2A_AR alone or with dual selectivity for the A_2A_AR and A_2B_AR. Cytokine production in CD8^+^ chimeric antigen receptor (CAR) T cells was increased, and both CD8^+^ and CD4^+^ CAR T cells were activated upon blocking the A_2A_AR.^7^ Thus, there is great interest in the discovery of novel A_2A_AR antagonists to act either centrally or peripherally, and new tools for ligand discovery are needed.

Various fluorescent probes have been developed over the past few years for characterizing ARs,^13^–^15^ and their use in fluorescence polarization (FP), fluorescence resonance energy transfer (FRET) and flow cytometry (FCM) has been proven to be feasible. Moreover, these ligands can provide a better understanding of receptor location, function and regulation. Most of the reports on fluorescent A_2A_AR antagonists used 5-amino-7-(3-(4-methoxy)phenylpropyl)-2-(2-furyl)pyrazolo[4,3-*e*]-1,2,4-triazolo[1,5-*c*]pyrimidine (SCH442416, **3**) as the pharmacophore due to the *p*-methoxyphenylpropyl side chain moiety of the antagonist, which served as the site for attachment of functionalized chains through an ether linkage.

Much of the ligand development for ARs now benefits from a detailed structural knowledge of the human (h)A_2A_AR.^16^–^20^ More than two dozen high-resolution X-ray crystallographic A_2A_AR structures with bound agonists or antagonists have been determined and used for the *in silico* screening of chemical libraries. A_2A_AR complexes with antagonist 4-(2-[7-amino-2-(2-furyl)[1,2,4]triazolo[2,3-*a*][1,3,5]triazin-5-yl-amino]ethyl)phenol (ZM241385, **2**) show that the heterocyclic pharmacophore binds to residues in the orthosteric binding pocket that also coordinates with the adenine moiety of AR agonists.^16^,^17^ Although no X-ray structure of the **3**–A_2A_AR complex has been determined, we used the **2**–A_2A_AR structure as the structural template for molecular modeling. The hypothetical docking pose of **3** and its AlexaFluor488 derivative **5** established most of the conserved interactions in the orthosteric binding site.^13^,^14^ The modeling also predicted stabilizing interactions of the tethered fluorophore with specific charged and H-bonding residues of the second extracellular loop (EL2).

Conjugate **5** was not optimal for fluorescent binding due to its moderate hA_2A_AR affinity (*K*_i_ = 111 nM).^13^ Also, its green light emission presents difficulties in fluorescence microscopy due to cell autofluorescence. Thus, there remains a need for A_2A_AR antagonist fluorescent probes of higher affinity and compatibility with microscopy. A BODIPY650/655 conjugate containing a secondary amine in the linking chain was reported to have a *K*_i_ value of 15 nM for the A_2A_AR, but its utility was not established.^13^ With this aim, we explored further the SAR of the distal region of this chemical series by varying the chain length of the spacer group and the terminal fluorophore in order to enhance the affinity, selectivity and photophysical properties.

## Results and discussion

**Scheme 1:**
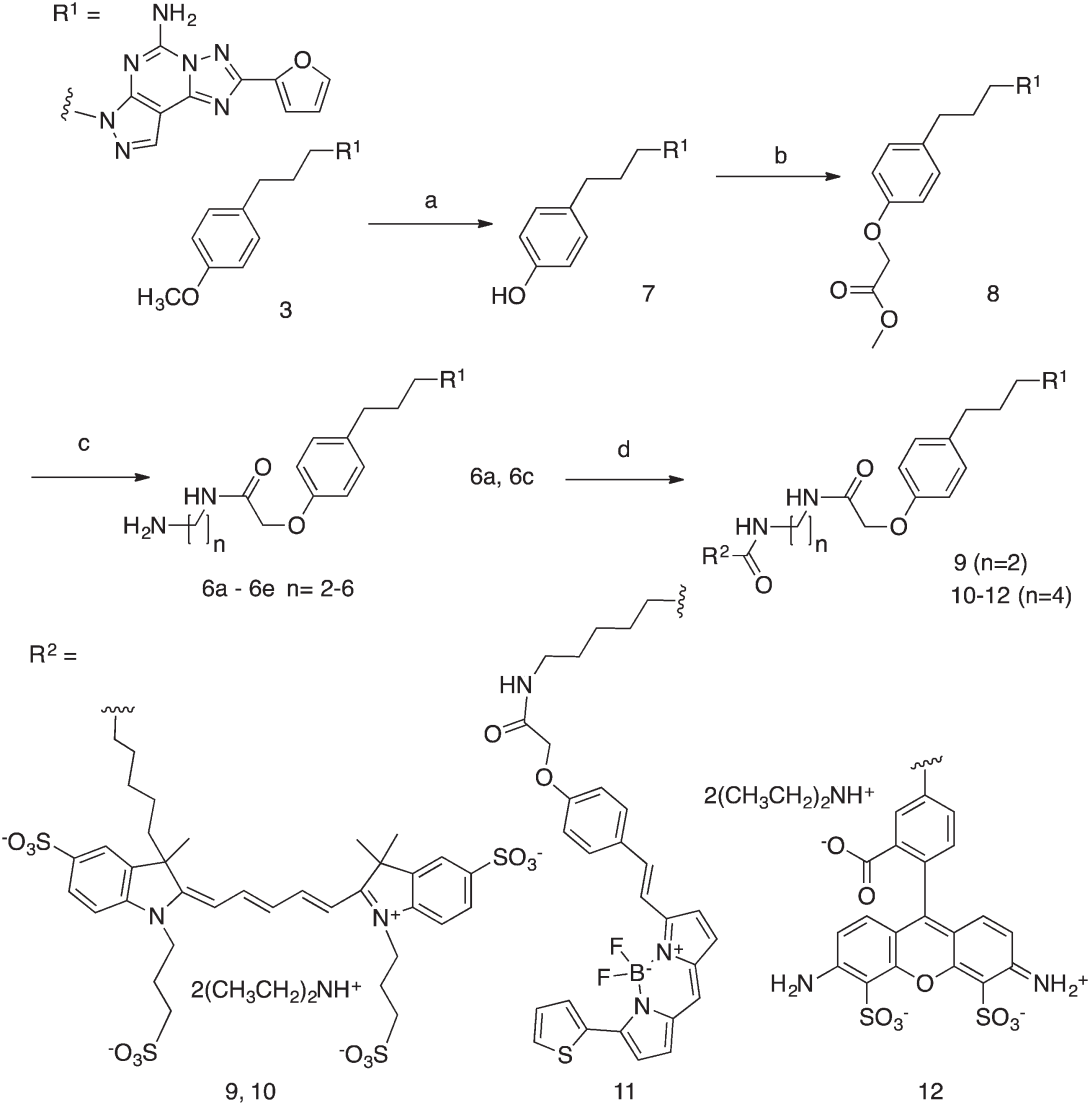
Synthesis of A_2A_AR antagonist functionalized congeners of **6** and their fluorescent derivatives **9–12**. (a) BBr_3_, CH_2_Cl_2_, rt, 4 h; (b) methyl 2-bromoacetate, Cs_2_CO_3_, MeOH, 40 °C, overnight; (c) diaminoalkane, MeOH (9 : 1 v/v), rt, overnight; (d) activated fluorophore (AlexaFluor647 *N*-hydroxysuccinimidyl ester for **9** and **10**, BODIPY 630/650 *N*-hydroxysuccinimidyl ester for **11**, AlexaFluor488 carboxylic acid, 2,3,5,6-tetrafluorophenyl ester for **12**), Et_3_N, DMF, rt, overnight.

We initially prepared a series of primary amine congeners of the A_2A_AR antagonist **3** by extending the *p*-methoxyphenylpropyl chain, which is predicted to lie outside the orthosteric binding site of the pharmacophore and be able to reach accessory sites.^13^ The congener **6a** (*n* = 2) was reported earlier,^13^ and we hypothesized that extension of the alkyl spacer could enhance the affinity or selectivity for this receptor, perhaps by establishing H-bond or electrostatic interactions with the polar residues in the EL region of the A_2A_AR. The synthesis of the pyrazolo[4,3-*e*][1,2,4]triazolo[1,5-*c*]pyrimidin-5-amine derivatives is shown in Scheme 1.

The synthesis of fluorescent ligands **9–12** was accomplished in 4 steps from commercially available **3**. The latter was demethylated to **7** by the action of BBr_3_ and then treated with an excess of methyl-2-bromoacetate (12 eq.) and Cs_2_CO_3_ as a base to provide ester **8** in 90% yield. Other methods, such as using NaH as the base with 1 eq. of methyl 2-bromoacetate in DMF, were attempted but led to the formation of di and tri-alkylated compounds. Amide synthesis was then performed by treatment of ester **8** with a series of alkyl diamines to give the amine congeners **6a–e**. Two of these amines, **6a** (*n* = 2) and **6c** (*n* = 4), were reacted with activated fluorophore moieties in DMF in the presence of Et_3_N to afford, after semi-preparative HPLC, compounds **9–12** in good yield (40–60%): cyanine 5 red fluorescent AlexaFluor647 (*n* = 2, **9**; *n* = 4, **10**), red fluorescent BODIPY630/650 (*n* = 4, **11**) and green fluorescent AlexaFluor488 (*n* = 4, **12**). Both fluorophores are commonly incorporated into ligand tools for chemical biology.^21^

**Fig. 1:**
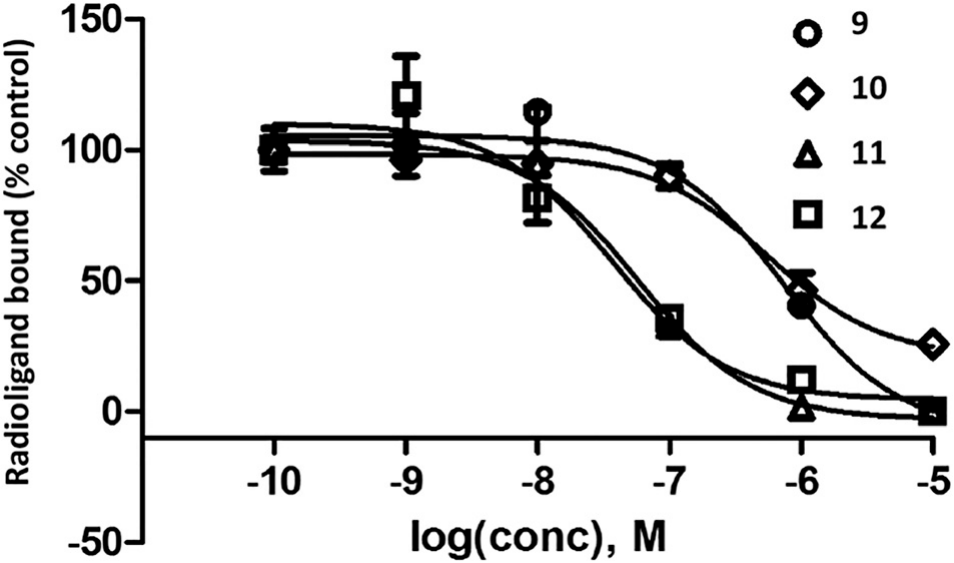
Antagonist radioligand ([^3^H]**2**, 1.0 nM) binding inhibition curves for the hA_2A_AR for four antagonist fluorescent conjugates. Compound numbers: MRS7322 **9**, MRS7395 **10**, MRS7396 **11** and MRS7416 **12**. Membranes from HEK-293 cells expressing the hA_2A_AR were used, and their incubation was performed for 1 h at 25 °C. Results are expressed as the mean ± SEM. The *K*_i_ values from three independent experiments are listed in Table 1.

The affinity of the amine congeners and other antagonists was measured using standard radioligand binding assays for hA_1_, A_2A_ and A_3_ARs (Table 1).^1^,^13^,^14^,^22^ Membranes of human embryonic kidney (HEK)293 cells expressing the AR of interest were used in the assay. Primary amine congeners **6a–6d** containing spacers of 2–5 methylenes were similar in terms of hA_2A_AR affinity (*K*_i_ 6–9 nM), with the butylamino congener **6c** displaying the highest A_2A_AR selectivity in the series. The affinity of this compound for the hA_3_AR was determined to be 6.0 μM, with only slightly higher hA_1_AR affinity. Homologation to 6 methylenes in compound **6e** lowered A_2A_AR affinity. Among the fluorescent conjugates of butylamino congener **6c**, *i.e.***10**, **11**, and **12**, the highest A_2A_AR affinity and selectivity were observed with the BODIPY630/650 fluorophore **11** and AlexaFluor488 **12** (Fig. 1). However, fluorescent **11** and **12** exhibited only a 4-fold weaker A_2A_AR affinity with respect to the parent amino derivative **6c**. Interestingly, AlexaFluor488 conjugate **12** was at least as potent in terms of A_2A_AR affinity as its shorter homologue **5**. No dependence of the affinity on the chain length was evident when comparing AlexaFluor647 conjugates **9** and **10**. Affinity for the mouse (m)ARs was also measured for the selected compounds by methods described,^22^ and **11** was particularly potent and selective for the mA_2A_AR (*K*_i_ = 2.1 nM), in contrast to **12** (*K*_i_ = 585 nM).

**Table 1:** AR binding affinity determined for a series of pyrazolo[4,3-*e*][1,2,4]triazolo[1,5-*c*]pyrimidin-5-amine derivatives (R^1^, as in Scheme 1). Human ARs, unless otherwise noted (m indicates mouse)

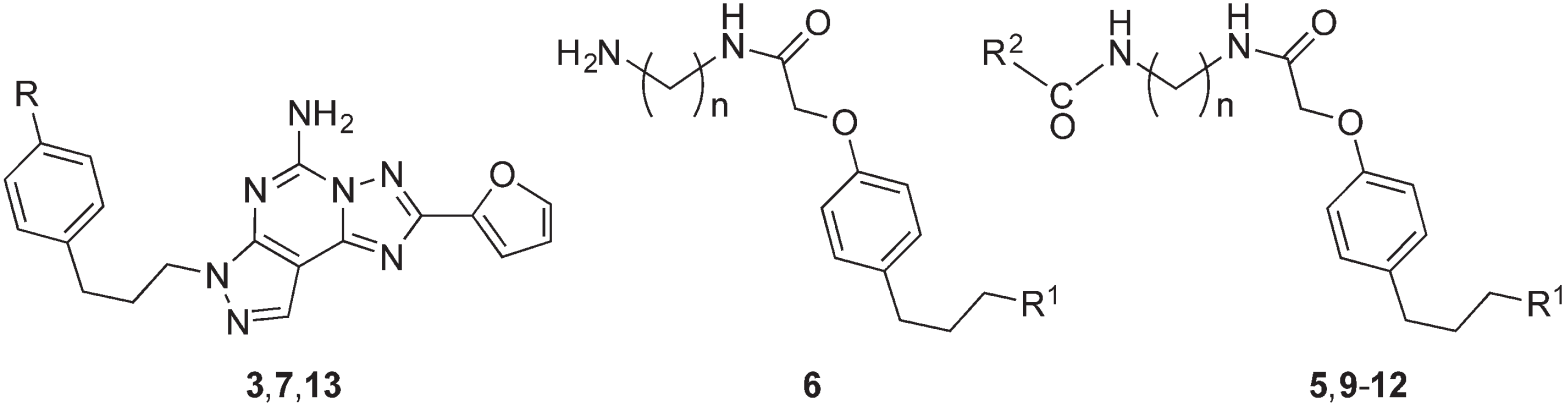				
		Affinity, *K*_i_, nM (or % inhib)		
Compd	Structure	A_1_^*a*^	A_2A_^*a*^	A_3_^*a*^
**3** ^*b*^	R = OCH_3_	*(35 ± 5%)* ^*c*^	4.1^*e*^	*(67 ± 1%)* ^*c*^
**5** ^*b*^	R^2^ = AlexaFluor488,^*d*^ *n* = 2	*(20 ± 3%)* ^*c*^	111 ± 16^*e*^	*(4 ± 2%)* ^*c*^
**6a** ^*b*^	*n* = 2	1270 ± 140^*c*^	6.8 ± 1.1^*e*^	3970 ± 120
**6b**	*n* = 3	1300 ± 350	9.29 ± 7.92	2170 ± 660
**6c**	*n* = 4	2390 ± 100	6.46 ± 1.63	5990 ± 2900
**6d**	*n* = 5	1910 ± 120	6.36 ± 3.58	656 ± 132
**6e**	*n* = 6	6500 ± 2830	22.8 ± 8.45	2070 ± 850
**7** ^*b*^	R = –OH	*(66 ± 2%)* ^*c*^	48 ± 28^*e*^	*(34 ± 3%)* ^*c*^
**9**	R^2^ = AlexaFluor647,^*d*^ *n* = 2	*(13 ± 6%)*,^*c*^ *(3 ± 1%)*^*c*^ (m)	332 ± 165, 458 ± 24 (m)	*(21 ± 3%)*,^*c*^ *(4 ± 1%)*^*c*^ (m)
**10**	R^2^ = AlexaFluor647,^*d*^ *n* = 4	*(20 ± 3%)* ^*c*^	295 ± 176	*(21 ± 2%)* ^*c*^
**11** ^*f*^	R^2^ = BODIPY630/650,^*d*^ *n* = 4	*(40 ± 3%)*,^*c*^ *(0%)*^*c*^ (m)	24.6 ± 17.6, 2.09 ± 0.16 (m)	*(33 ± 5%)*,^*c*^ *(2 ± 2%)*^*c*^ (m)
**12** ^*f*^	R = AlexaFluor488,^*d*^ *n* = 4	1680 ± 470, *(0%)*^*c*^ (m)	30.3 ± 4.9, 585 ± 73 (m)	*(32 ± 3%)*,^*c*^ *(5 ± 2%)*^*c*^ (m)
**13** ^*f*^	R = –OCH_2_-Ph-*p*-SO_3_H	4190 ± 750, *(21 ± 4%)*^*c*^ (m)	6.24 ± 2.42, 64.1 ± 5.3 (m)	2660 ± 1250, *(4 ± 1%)*^*c*^ (m)

^*a*^Competition radioligand binding assays were conducted with membranes prepared from HEK-293 cells expressing recombinant A_1_, A_2A_, or A_3_ARs (human) unless otherwise noted. Their incubation was performed for 1 h at 25 °C. The radioligands used were: A_1_AR, [^3^H]8-cyclopentyl-1,3-dipropylxanthine ([^3^H]DPCPX, 0.5 nM) **14**; A_2A_AR, [^3^H]ZM241385 **2** (1.0 nM) or from published data^13^ (as noted) [^3^H]2-[*p*-(2-carboxyethyl)phenyl-ethylamino]-5′-*N*-ethylcarboxamidoadenosine ([^3^H]CGS21680, 10 nM) **15**; A_3_AR, [^125^I]*N*^6^-(4-amino-3-iodobenzyl)adenosine-5′-*N*-methyluronamide ([^125^I]I-AB-MECA, 0.2 nM) **16**. Nonspecific binding was determined using 10 μM 8-[4-[[[[(2-aminoethyl)amino]carbonyl]methyl]oxy]phenyl]-1,3-dipropylxanthine (XAC) **17** (A_1_AR and A_2A_AR) or 10 μM adenosine-5′-*N*-ethyluronamide (NECA) **18** (A_3_AR). HEK-293 cells expressing recombinant mA_1_, mA_2A_, or mA_3_ARs were used. Values are expressed as the mean ± SEM from 3 independent experiments. The cell lines were from the American Type Culture Collection (ATCC, Manassas, VA), and the cDNA for the ARs was obtained from cdna.org.

^*b*^Data from Kumar *et al.* and Kecskés *et al.*^13^,^14^

^*c*^Percent inhibition at 10 μM.

^*d*^Fluorophore moiety, as shown in Scheme 1 (R^2^).

^*e*^Using [^3^H]**16** as the radioligand.

^*f*^ **11**, MRS7396; **12**, MRS7416; **13**, MRS7352.

**Fig. 2:**
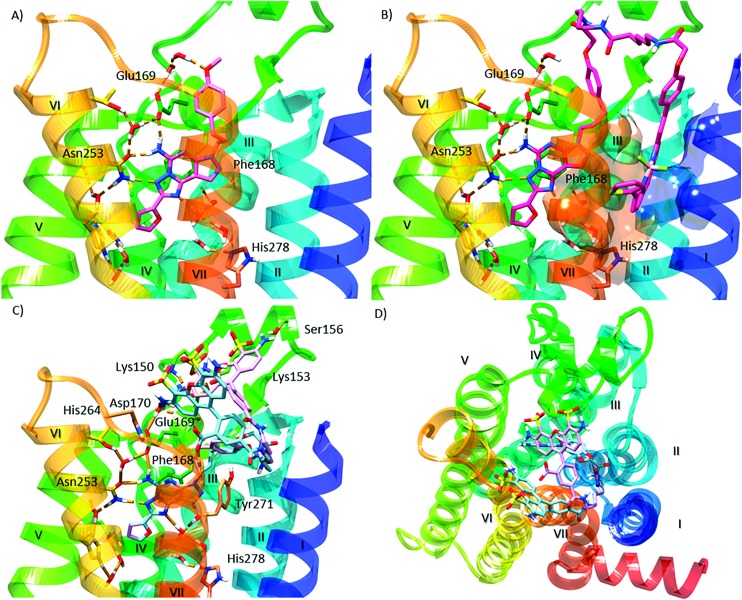
Molecular modeling of antagonist binding to the hA_2A_AR. Details of the binding site of the X-ray structure of the receptor modeled with various ligands docked: (A) known antagonist **3**, with the retention of a subset of water molecules found in the high resolution A_2A_AR structure;^17^ (B) the BODIPY630/650-labeled antagonist **11**, showing the most energetically favorable orientation of the terminal fluorophore chain; side (C) and top (D) views of the two possible orientations of the fluorophore group of derivative **12**. Residues establishing polar (dashed orange lines) and π–π interactions with the docked ligands are represented as sticks. Aromatic residues establishing hydrophobic contacts with the terminal fluorophore of compound **11** are represented as transparent surfaces with colors matching the corresponding TM domain. Non-polar hydrogen atoms are omitted.

We performed molecular modeling analysis to identify possible binding modes of fluorescent conjugates **11** and **12** using the high resolution **2**-A_2A_AR X-ray structure (PDB ID: ; 4EIY).^17^ We first docked reference compound **3** at the hA_2A_AR by retaining several water molecules observed in the X-ray structure as described (ESI†). In the corresponding docking pose (Fig. 2A), the pyrazolotriazolopyrimidine core of **3** docked in the orthosteric binding site similar to the triazolopyrimidine core of **2**, with a π–π stacking interaction of the aromatic core with F168 (EL2), and a H-bonding network with N253 (6.55, using standard GPCR notation^23^) and E169 (EL2). The methoxy-phenyl substituent of **2** pointed toward the extracellular (EC) side of the receptor and interacted with the side chain of Glu169 (EL2) through a water molecule. The binding mode of **3** described above was validated using 30 ns molecular dynamics (MD) simulation. During the simulation (Video S1, replica analysis reported in Table S1†), the methoxy-phenyl moiety folded toward transmembrane domain 2 (TM2) and established a π–π stacking interaction with Y271 (7.36), while the aromatic core maintained the H-bond network observed in the initial docking pose. Notably, the most energetically favored ligand–protein complex featured the same interaction pattern described above (Fig. S1†).

The potent fluorescent conjugate **11** was then docked in the A_2A_AR structure (Fig. 2B) with the same water molecules retained as in the **3**–A_2A_AR complex.^17^ The pyrazolotriazolopyrimidine core established the same interactions observed for **3** in the orthosteric binding site. The fluorophore linker pointed toward the EC side and folded back toward the TM bundles by directing the fluorophore group to an aromatic pocket at the interface between TM1 and TM7 (transparent surface in Fig. 2B). To explore other orientations of the linker at the receptor's EC side, we docked compound **6c**, the amino precursor of **11**, at the hA_2A_AR. To sample all the possible H-bond acceptor/donor partners on the EC side of the receptor, we removed water molecules interacting with E169 during the docking. We then clustered the binding modes obtained according to the linker orientation. This selection resulted in two alternative binding modes (Fig. S2†) that were subsequently subjected to MD validation (30 ns of simulation run in triplicate for each binding mode, see Table S1†). In the most energetically favored docking mode (hereby referred to as “BM1”, orange carbon sticks in Fig. S2,† docking score = –12.077 kcal mol^–1^), the tail was oriented toward TM4 and TM5 with the amide moiety establishing a H-bond with the side chain of E169, while the terminal amine group H-bonded with the backbone of E169 (EL2) and the side chain of K150 (EL2). This latter H-bond was expected to be unstable in a dynamic environment due to competition in the formation of a salt-bridge between K150 and D170 (EL2). In the alternative binding mode (BM2, green carbon sticks in Fig. S2,† docking score = –10.994 kcal mol^–1^), the tail was oriented toward TM1 and TM2 and did not establish additional interactions. MD trajectory analysis revealed that BM1 achieved ligand–protein complexes that were more energetically favored (data not shown). Fig. S3† depicts the two **11**–A_2A_AR complexes with the most favorable ligand–protein interaction energy (IE, values differed by less than 2 kcal mol^–1^ and were considered equivalent) obtained for BM1 that features different orientations of the tail. We therefore searched for hydrophobic and aromatic regions in the proximity of the terminal amine moiety to investigate the compatibility of these orientations with the fluorophore insertion. Specifically, we searched for receptor regions rich in hydrophobic and aromatic residues at 5, 13, and 14 Å from the nitrogen atom of the terminal alkylamino group of **6c** (Fig. S3B†). The choice of distances reflected the analysis of the N-group distance to aromatic rings in both the docked and the energy minimized three-dimensional structures of **11** (Fig. S3A†). Of the two possible orientations of **11**, only one displayed hydrophobic and aromatic residues at distances compatible with the insertion of the aromatic fluorophore. Notably, the aromatic/hydrophobic region is located at the interface between TM1 and TM7 (Fig. S3C†), thus suggesting the same orientation of the ligand as observed from the docking analysis. Nonetheless, we cannot exclude that **11** might explore different regions on the receptor's EC side.

The fluorescent conjugate **12** (considered as the species carrying a –2 net charge) was docked in the A_2A_AR structure by following the same procedure described for compound **11** (ESI†). The docking output suggested two equally plausible orientations of the fluorophore (Fig. 2C and D). In one docking pose (BM1, cyan carbon sticks in Fig. 2C, docking score = –11.490 kcal mol^–1^), the fluorophore group was projected toward EL3. In the alternative binding mode (BM2, purple carbon sticks, docking score = –11.279 kcal mol^–1^), the fluorophore group interacted with the residues in EL2 and EL3. The interaction pattern of the core was the same as those for the other members of this chemical series: H-bonds with N253 and E169 (EL2); π–π stacking with H252 (6.52), F168 (EL2), and Y271 (7.36). In the MD simulation starting from BM1 (30 ns run in triplicate, see Table S1†) the fluorophore group and the linker fluctuated on the EC surface of the receptor without engaging in specific interactions except for a H-bond involving the fluorophore carboxylate moiety (data not shown). On the other hand, the simulations starting from BM2 (30 ns, replica analysis in Table S1†) achieved more energetically favored ligand–protein complexes. The simulations returned the ligand–protein complex characterized by the lowest IE value for all trajectories and converged in a unique binding mode (Fig. S4†) featuring the fluorophore group stacked between EL2 and EL3. In such a conformation, the ligand established a tight network of H-bonds and salt-bridges between charged residues in EL2 and EL3 and the polar/charged counterparts in the fluorophore moiety. In particular, during the MD simulation (visualization of run 3 trajectory selected as an example, Video S2†) the sulfonic groups established salt bridges with K153 (persistent) and K150 (intermittent) in EL2, one of the amine moieties interacted with the sidechain of E169 (EL2), and the carboxylate moiety of the ligand replaced E169 (EL2) in the salt bridge with H264 (EL3). Moreover, for most of the total simulation time, the pyrazolotriazolopyrimidine core maintained its interaction pattern with N253 (6.55) and F168 (EL2), while the linker between the core and the fluorophore group was anchored to TM7, EL2 and TM2 through H-bond interactions with the backbone of S67 (2.65) and the sidechains of Q157 (EL2) and Y271 (7.36), respectively (Video S2†).

Thus, both **11** and **12** are bitopic in the sense that each bridged two separate domains of the A_2A_AR, *i.e.* the orthosteric binding site that is well defined in X-ray structures^16^,^17^ and an additional domain. The BODIPY630/650 fluorophore of **11** is predicted to be buried in a hydrophobic region, while AlexaFluor488 of **12** associates with the hydrophilic ELs. The bitopic nature of these conjugates does not necessarily imply allosteric modulation of the orthosteric action,^28^ which is unexplored in this series.

In order to further explore the ligand interactions with the EC surface, we prepared an aryl sulfonate **13**, which contained a terminal group capable of multiple polar interactions (Scheme S1, ESI†). This terminal phenylsulfonic acid moiety also would allow for π–π interactions with aromatic residues. A further benefit of incorporating a sulfonate group is that it carries a permanent negative charge at physiological pH and would prevent penetration into the blood brain barrier,^22^ a useful characteristic for a pharmacological probe for *in vivo* studies.^24^**13** was potent and selective for binding to the h and mA_2A_ARs, with 671- and 426-fold selectivity compared to the hA_1_AR and hA_3_AR, respectively. **13** binding to the mA_1_AR and mA_3_AR was insignificant.

**Fig. 3:**
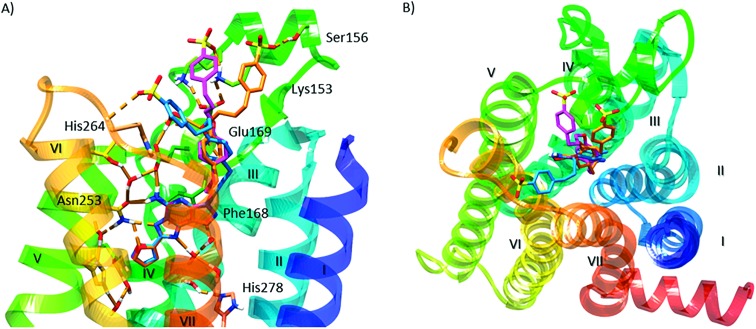
Modeling of antagonist **13** binding to the hA_2A_AR: side (A) and top (B) views of the three possible orientations (BM1, blue; BM2, magenta; BM3, orange) of the *p*-sulfophenyl tail. Residues establishing polar (dashed orange lines) and π–π interactions with the docked ligands are represented as sticks. Non-polar hydrogen atoms are omitted.

The binding of **13** was modeled using the same docking procedure and MD validation described for **6c**. Fig. 3 depicts the three alternative binding modes of **13**. In the most energetically favorable pose (BM1, blue carbon sticks) the sulfophenyl ring established a π–π stacking interaction with H264 (EL3) and the sulfonic group engaged in an H-bond interaction with the residue backbone. In the other two docking poses (BM2 and BM3, magenta and orange carbon sticks, respectively) the sulfophenyl ring established a π–cation interaction with K153 (EL2) and the sulfonic group interacted with the side chain of either K153 (EL2; BM2, magenta) or S156 (EL2; BM3, orange). During the MD simulation, the ligand's sulfophenyl tail fluctuated considerably (high averaged root mean square deviation values, Table S1†), thus demonstrating the instability of the interaction pattern predicted in the initial docking poses. On the other hand, the three different initial poses converged in a unique binding mode, as the ligand–protein complexes with the lowest IE value returned by each different binding mode featured the same conformation (Fig. S5†). In particular, the 7-phenylpropyl ring moved toward TM7 to establish a π–π stacking interaction with Y271 (7.36).

Two representative derivatives were shown to be A_2A_AR antagonists in a cyclic AMP assay (Fig. S6†). The activation curve for the known agonist **15** right-shifted in a parallel manner at fixed concentrations of **11** and **13**. The EC_50_ of **15** shifted from 0.89 ± 0.17 nM to 128 ± 35 nM in the presence of **11** (1000 nM) and to 10.2 ± 2.3 nM in the presence of **13** (100 nM). Off-target binding activities at 45 diverse receptors were determined by PDSP^25^ for selected compounds: **6c** and **13** (ESI†). The only *K*_i_ values below 10 μM were: **6c**, 3.36 μM, 5HT_2A_ serotonin receptor; 1.92 μM, 5HT_2B_ receptor. Thus, the primary amine precursor of the fluorescent conjugates **10–12** was not promiscuous in its interaction with other proteins, *i.e.* this chemical series is not associated with pan-assay interference compounds.^26^

**Fig. 4:**
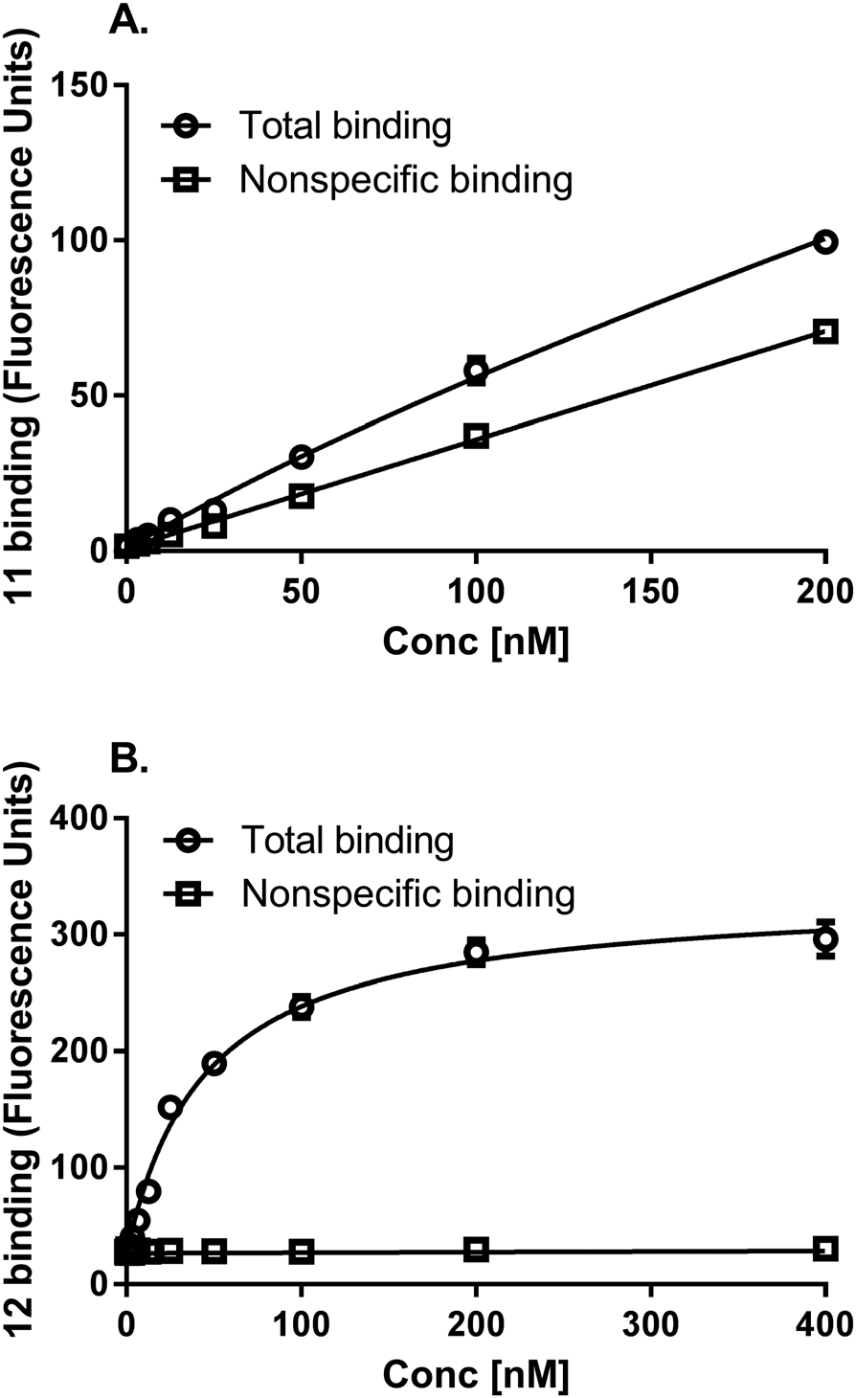
Saturation of fluorescent antagonists **11** (A) and **12** (B) in hA_2A_AR-expressing HEK-293 cells measured using flow cytometry following a 1 h incubation at 37 °C. Non-specific binding was determined with 10 μM **3**. Results are expressed as the mean ± SEM. The *K*_d_ value calculated from Fig. 4B is listed in the text.

**Fig. 5:**
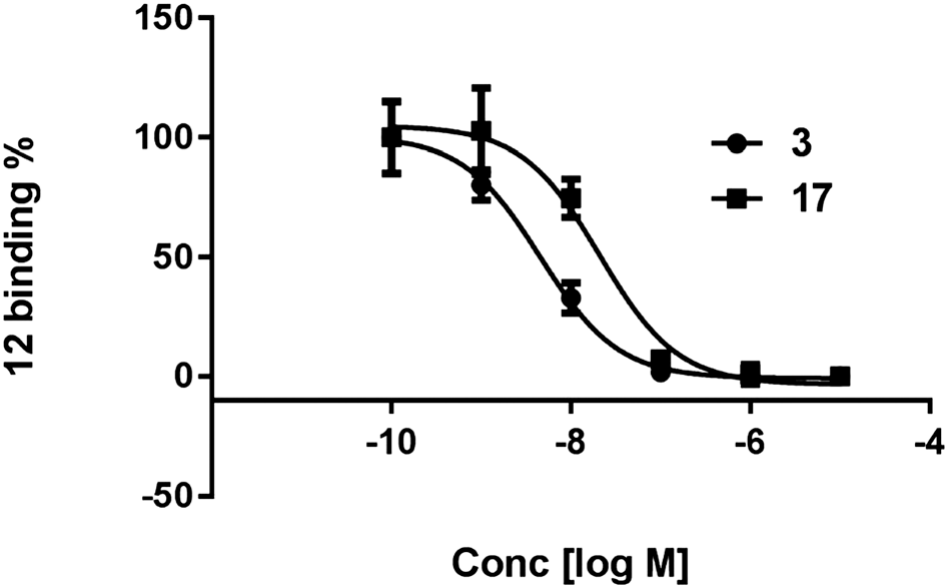
Inhibition of the specific binding of fluorescent antagonist **12** (10 nM, 1 h incubation at 37 °C) in hA_2A_AR-expressing HEK-293 cells measured using flow cytometry. Competing antagonists were: nonxanthine **3** and xanthine **17**. Non-specific binding was determined with 10 μM **3**. Results are expressed as the mean ± SEM. The *K*_i_ values are listed in the text.

Compounds **11** and **12** were tested as fluorescent tracers for flow cytometry of HEK-293 cells expressing the hA_2A_AR (Fig. 4A). **11** proved to have high nonspecific binding, with a low level of specific binding amounting to <25% of the total and therefore was not optimal for flow cytometric analysis. Thus, **11** tended to bind non-specifically to hydrophobic membranes by undetermined mechanisms. However, the more hydrophilic **12** displayed low nonspecific binding by this method, and its binding was saturable with a *K*_d_ value of 45.4 nM (Fig. 4B). For inhibition studies, the cells were co-incubated for one hour prior to flow cytometry with fluorescent probe **12** (10 nM) and a test antagonist. The binding of **12** was inhibited by two known A_2A_AR antagonists, nonxanthine **3** and xanthine **17**, with the expected range of potency.^27^ The *K*_i_ values were 3.8 and 17.2 nM (Fig. 5), respectively, which corresponded closely to the reported values of 4.1 and 18 nM at hA_2A_AR.^1^,^13^ Agonist inhibition of the competitive binding of **12** was complex (ESI†).

## Conclusions

We succeeded in identifying useful A_2A_AR probes by systematically varying the chain length of amine-functionalized congeners of a potent antagonist and coupling the primary amine having an optimal length to various fluorophores. We successfully enhanced their A_2A_AR affinity compared to known fluorescent A_2A_AR ligands.^15^ Conjugates **11** and **12** were potent and selective antagonist probes for the A_2A_AR, but **12** was more promising for characterization of the hA_2A_AR in whole cells by flow cytometry. Molecular modeling suggested that the fluorophore of **11** interacted with hydrophobic regions between TMs. However, the hydrophilic fluorophore of **12** was coordinated to the ELs, consistent with its lower overall hydrophobicity and more favorable whole cell binding characteristics compared to **11** under the present conditions. Evaluation of **11** and **12** by confocal microscopy studies remains to be performed. Thus, we have introduced antagonist ligands displaying high A_2A_AR affinity and selectivity that may serve as versatile tools to better study this receptor.

## Conflict of interest

The authors declare no competing interests.

## Abbreviations

AN: Acetonitrile
AR: Adenosine receptor
BODIPY: 4,4-Difluoro-4-bora-3a,4a-diaza-*s*-indacene
CNS: Central nervous system
DMF: *N*,*N*-Dimethylformamide
EDC: *N*-(3-Dimethylaminopropyl)-*N*′-ethylcarbodiimide
HEK: Human embryonic kidney
IE: Interaction energy
NECA: 5′-*N*-Ethylcarboxamidoadenosine
PD: Parkinson's disease
TBAP: Tetrabutylammonium dihydrogen phosphate
THF: Tetrahydrofuran
TLC: Thin layer chromatography
TM: Transmembrane domain

## Supplementary Material

Supplementary information

Supplementary information

Supplementary information

Supplementary movie

Supplementary movie
