# Supplementary material for: Bitopic fluorescent antagonists of the A2A adenosine receptor based on pyrazolo[4,3-e][1,2,4]triazolo[1,5-c]pyrimidin-5-amine functionalized congeners
Source: Medchemcomm. 2017 Jun 22;8(8):1659–67. doi: 10.1039/c7md00247e (PMC5729930; doi:10.1039/c7md00247e)
Supplement: Supplementary file 3 [file MD-008-C7MD00247E-s003.pdf]

Electronic Supplementary Material (ESI) for MedChemComm.
This journal is © The Royal Society of Chemistry 2017

Supporting information

Bitopic Fluorescent Antagonists of the A2A Adenosine Receptor Based on Pyrazolo[4,3-*e*][1,2,4]triazolo[1,5-*c*]pyrimidin-5-amine Functionalized Congeners

Romain Duroux, Antonella Ciancetta, Philip Mannes, Jinha Yu, Shireesha Boyapati, Elizabeth Gizewski, Said Yous, Francisco Ciruela, John A. Auchampach, Zhan-Guo Gao, and Kenneth A. Jacobson

Contents

**Synthetic Methods**	S1-S7
**Molecular Modeling Methods**	S7-S9
**Molecular Modeling Results**	S7-S14
**Pharmacological Results**	S15-S16
**Off-target interactions for selected compounds**	S17
**Representative NMR and Mass Spectra and HPLC Analysis**	S18-S28
**Visible and UV spectral data (9)**	S29

Synthetic methods

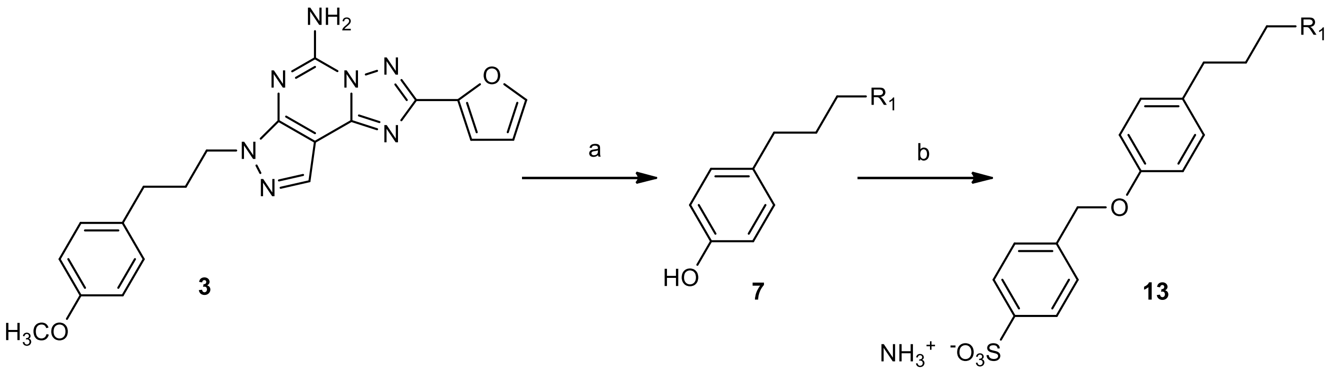

Scheme S1. (a) BBr_3, CH_2Cl_2, rt, 4h; (b) sodium 4-(bromomethyl)benzenesulfonate, NaH, DMF, rt, 2h, 42%.

Chemical Synthesis. Materials and Instrumentation. Compound **3** (Tocris Bioscience, Ellisville, MO), Alexa Fluor® 647 NHS Ester (tri-potassium salt, ThermoFisher Scientific, Ref. A20006), BODIPY® 630/650-X NHS Ester (ThermoFisher Scientific, Ref. D10000) and Alexa Fluor® 488 Carboxylic Acid, 2,3,5,6-Tetrafluorophenyl Ester, 5-isomer (di-triethylamine salt, ThermoFisher Scientific, Ref. A30005)

were obtained from the commercial sources specified in the parenthesis next to its name. All other reagents and solvents were purchased from Sigma-Aldrich (St. Louis, MO). NMR spectra were recorded on a Bruker 400 MHz spectrometer. Chemical shifts are given in ppm (δ), calibrated to the residual solvent signals or TMS. TLC analysis was carried out on glass sheets precoated with silica gel F 254 (0.2 mm) from Aldrich and spots were examined under ultraviolet light at 254 nm. Purification of final fluorescent compounds was performed by preparative HPLC with CH_3CN/H_2O as mobile phase (column A: Luna 5 µm C18(2) 100 Å, LC column 250 mm × 21.2 mm, flow rate of 5 mL/min; column B: Eclipse XDB-C18, 5 µm, 4.6 mm × 250 mm, flow rate of 5 mL/min). Column chromatography was performed on silica gel (40−63 µm, 60 Å). High resolution mass (HRMS) measurements were performed on a proteomics optimized Q-TOF-2 (Micromass-Waters). The purity of final derivatives was checked using a Hewlett-Packard 1100 HPLC equipped with a Zorbax SB-Aq 5 µm analytical column (50 × 4.6 mm; Agilent Technologies Inc., Palo Alto, CA). The mobile phase was as follows: linear gradient solvent system, 5 mM TBAP (tetrabutylammonium dihydrogen phosphate) – CH_3CN from 100:0 to 0:100 in 15 min; the flow rate was 0.5 mL/min. All derivatives tested for biological activity showed >95% purity by HPLC analysis with detection at 254 nm for molecules without fluorescent moieties and at 488 nm, 640 nm or 647 nm depending on the fluorescent ligands.

General procedure for synthesis of compounds **6a** – **6e**, by aminolysis of ester **8**:

Compound **8** (1 eq., 0.047 mmol) was dissolved in a mixture of the corresponding dialkylamine and MeOH (7 mL, 1:9, v/v) and stirred overnight at room temperature before concentrated to dryness. The resulting residue was purified by silica gel column chromatography (20:78:2, MeOH:CH_2Cl_2:aq.NH_3, v/v/v) to afford the desired product.

2-(4-(3-(5-Amino-2-(furan-2-yl)-7*H*-pyrazolo[4,3-*e*][1,2,4]triazolo[1,5-*c*]pyrimidin-7-yl)propyl)phenoxy)-*N*-(2-aminoethyl)acetamide **6a**.

Compound **8** (1 eq., 12 mg, 0.0268 mmol) was dissolved in a mixture of ethylenediamine and MeOH (2 mL, 1:9, v/v). After stirring at room temperature overnight, the reaction mixture was concentrated to dryness, and the resulting residue was purified by silica gel column chromatography (20:78:2 MeOH:CH_2Cl_2:aq.NH_3, v/v/v) to afford a white solid (8 mg, 63 %) as a white solid. ^{1}H NMR (MeOD-*d*4, δ ppm) 8.10 (s, 1H), 7.77 (d, *J* = 0.7 Hz and *J* = 1.7 Hz, 1H), 7.26 (d, *J* = 0.7 Hz and *J* = 3.4 Hz, 1H), 7.10 (d, 2H, *J* = 8.7 Hz), 6.84 (d, 2H, *J* = 8.7 Hz), 6.68 (dd, 1H, *J* = 1.6 Hz and *J* = 3.2 Hz), 4.44 (s, 2H), 4.38 (t, 2H, *J* = 6.7 Hz), 3.33 (t, 2H, *J* = 6.2 Hz), 2.77 (t, 2H, *J* = 6.2 Hz), 2.62 (t, 2H, *J* = 6.2 Hz), 2.26-2.23 (m, 2H). ESI-HRMS calculated for $C_{23}H_{26}N_9O_3$ $[M + H]^+$, 476.2160; Calcd. 476.2159. HPLC purity 98% (R_t = 6.9 min).

2-(4-(3-(5-Amino-2-(furan-2-yl)-7H-pyrazolo[4,3-e][1,2,4]triazolo[1,5-c]pyrimidin-7-yl)propyl)phenoxy)-*N*-(3-aminopropyl)acetamide **6b**.

Compound **8** (1 eq., 7 mg, 0.0157 mmol) was dissolved in a mixture of 1,3-diaminopropane and MeOH (2 mL, 1:9, v/v). After stirring at room temperature overnight, the reaction mixture was concentrated to dryness, and the resulting residue was purified by silica gel column chromatography (20:78:2 MeOH:CH_2Cl_2:aq.NH_3, v/v/v) to afford a white solid (4.5 mg, 59 %). ^{1}H NMR (MeOD-*d*4, δ ppm) 8.10 (s, 1H), 7.77 (d, *J* = 1.7 Hz, 1H), 7.26 (d, *J* = 3.2 Hz, 1H), 7.10 (d, 2H, *J* = 8.7 Hz), 6.84 (d, 2H, *J* = 8.7 Hz), 6.68 (dd, 1H, *J* = 1.6 Hz and *J* = 3.2 Hz), 4.43 (s, 2H), 4.38 (t, 2H, *J* = 6.7 Hz), 3.33 (t, 2H, *J* = 6.2 Hz), 2.68 (t, 2H, *J* = 6.2 Hz), 2.62 (t, 2H, *J* = 6.2 Hz), 2.26-2.23 (m, 2H), 1.74-1.67 (m, 2H). ESI-HRMS calculated for $C_{24}H_{28}N_9O_3$ $[M + H]^+$, 490.2321; Calcd. 490.2315. HPLC purity 99% (R_t = 6.9 min).

2-(4-(3-(5-Amino-2-(furan-2-yl)-7H-pyrazolo[4,3-e][1,2,4]triazolo[1,5-c]pyrimidin-7-yl)propyl)phenoxy)-*N*-(4-aminobutyl)acetamide **6c**.

Compound **8** (1 eq., 21 mg, 0.047 mmol) was dissolved in a mixture of putrescine and MeOH (7 mL, 1:9, v/v) and stirred overnight at room temperature before concentrated to dryness. The resulting residue was purified by silica gel column chromatography (20:78:2 MeOH:CH_2Cl_2:aq.NH_3, v/v/v) to afford a white solid (22 mg, 94 %). ^{1}H NMR (MeOD-d_4, δ ppm) 8.10 (s, 1H), 7.78 (d, J = 1.7 Hz, 1H), 7.26 (d, J = 0.7 Hz and J = 3.4 Hz, 1H), 7.10 (d, 2H, J = 8.7 Hz), 6.84 (d, 2H, J = 8.7 Hz), 6.68 (dd, 1H, J = 1.6 Hz and J = 3.2 Hz), 4.42 (s, 2H), 4.38 (t, 2H, J = 6.7 Hz), 3.33 (t, 2H, J = 6.2 Hz), 2.90 (t, 2H, J = 6.2 Hz), 2.62 (t, 2H, J = 6.2 Hz), 2.26-2.23 (m, 2H), 1.62-1.61 (m, 4H). ESI-HRMS calculated for $C_{25}H_{30}N_9O_3$ $[M + H]^+$, 504.2476; Calcd. 504.2472. HPLC purity 97% (R_t = 7.4 min).

2-(4-(3-(5-amino-2-(furan-2-yl)-7H-pyrazolo[4,3-e][1,2,4]triazolo[1,5-c]pyrimidin-7-yl)propyl)phenoxy)-*N*-(5-aminopentyl)acetamide **6d**.

Compound **8** (1 eq., 10 mg, 0.0224 mmol) was dissolved in a mixture of cadaverine and MeOH (5 mL, 1:9, v/v) and stirred overnight at room temperature before concentrated to dryness. The resulting residue was purified by silica gel column chromatography (20:78:2 MeOH:CH_2Cl_2:aq.NH_3, v/v/v) to afford a white solid (7.5 mg, 65 %). ^{1}H NMR (MeOD-d_4, δ ppm) 8.10 (s, 1H), 7.78 (d, J = 0.7 Hz and J = 1.7 Hz, 1H), 7.26 (d, J = 0.7 Hz and J = 3.4 Hz, 1H), 7.10 (d, 2H, J = 8.7 Hz), 6.83 (d, 2H, J = 8.7 Hz), 6.68 (dd, 1H, J = 1.6 Hz and J = 3.2 Hz), 4.42 (s, 2H), 4.38 (t, 2H, J = 6.8 Hz), 3.27 (t, 2H, J = 7.2 Hz), 2.74-2.67 (m, 4H), 2.62 (t, 2H, J = 7.2 Hz), 2.26-2.23 (m, 2H), 1.61-1.51 (m, 4H). ESI-HRMS calculated for $C_{26}H_{32}N_9O_3$ $[M + H]^+$, 518.2631; Calcd. 518.2628. HPLC purity 96% (R_t = 7.8 min).

2-(4-(3-(5-Amino-2-(furan-2-yl)-7H-pyrazolo[4,3-e][1,2,4]triazolo[1,5-c]pyrimidin-7-yl)propyl)phenoxy)-*N*-(6-aminohexyl)acetamide **6e**.

Compound **8** (1 eq., 7 mg, 0.0157 mmol) was dissolved in a mixture of 1,6-diaminohexane and MeOH (2 mL, 1:9, v/v) and stirred overnight at room temperature before concentrated to dryness. The resulting residue was purified by silica gel column chromatography (20:78:2 MeOH:CH_2Cl_2:aq.NH_3, v/v/v) and to afford a white solid (1.5 mg, 20 %). ^{1}H NMR (MeOD-d_4, δ ppm): 8.10 (s, 1H), 7.78 (d, J = 0.7 Hz and J = 1.7 Hz, 1H), 7.26 (d, J = 0.7 Hz and J = 3.4 Hz, 1H), 7.10 (d, 2H, J = 8.7 Hz), 6.84 (d, 2H, J = 8.7 Hz), 6.68 (dd, 1H, J = 1.6 Hz and J = 3.2 Hz), 4.42 (s, 2H), 4.38 (t, 2H, J = 6.8 Hz), 3.27 (t, 2H, J = 7.2 Hz), 2.91 (t, 2H, J = 7.2 Hz), 2.62 (t, 2H, J = 7.2 Hz), 2.26-2.23 (m, 2H), 1.68-1.66 (m, 2H), 1.64-1.62 (m, 2H), 1.61-1.58 (m, 4H). ESI-HRMS calculated for $C_{27}H_{34}N_9O_3$ $[M + H]^+$, 532.2776; Calcd. 532.2785. HPLC purity 96% (R_t = 7.5 min).

4-(3-(5-Amino-2-(furan-2-yl)-7H-pyrazolo[4,3-e][1,2,4]triazolo[1,5-c]pyrimidin-7-yl)propyl)phenol **7**.

To a solution of 2-(furan-2-yl)-7-(3-(4-methoxyphenyl)propyl)-7H-pyrazolo[4,3-e][1,2,4]triazolo[1,5-c]pyrimidin-5-amine (**3**, 1 eq., 90 mg, 0.231 mmol) in DCM (12 mL) was added dropwise BBr_3 (5 eq., 1 M in CH_2Cl_2, 1.18 mL, 1.18 mmol) at 0 °C. The mixture was stirred for 4 h at room temperature, hydrolyzed carefully with MeOH at 0 ˚C and evaporated *in vacuo* to afford a brown solid, which was used without further purification in the next step (83 mg, 95 %). ^{1}H NMR (MeOD-d_4, δ ppm) 8.12 (s, 1H), 7.78 (d, J = 1.1 Hz, 1H), 7.25 (d, J = 3.1 Hz, 1H), 6.92-7.10 (d, J = 8.5 Hz, 2H), 6.66 (d, J = 1.9 Hz, 1H), 6.61-6.65 (d, J = 8.5 Hz, 2H), 4.37 (t, J = 7.0 Hz, 2H), 2.52 (t, J = 7.0 Hz, 2H), 2.16-2.25 (m, 2H). ESI-HRMS calculated for $C_{19}H_{18}N_7O_2$ $[M + H]^+$, 376.1532; Calcd. 376.1522.

Methyl 2-(4-(3-(5-amino-2-(furan-2-yl)-7H-pyrazolo[4,3-e][1,2,4]triazolo[1,5-c]pyrimidin-7-yl)propyl)phenoxy)acetate **8**.

To a suspension of **7** (1 eq., 18 mg, 0.048 mmol) in MeOH (3 mL) was added cesium carbonate (5 eq., 78.1 mg, 0.24 mmol). The mixture was stirred for 1 h at 40 °C and then methyl bromoacetate (12 eq., 0.055 mL, 0.58 mmol) was added. The mixture was stirred overnight at 40 °C and then concentrated *in vacuo*. The crude product was purified by silica gel column chromatography (DCM/MeOH : 99/1) to afford a white solid (12 mg, 90 %). ^{1}H NMR (MeOD-d_4, δ ppm) : 8.21 (s, 1H), 7.65 (d, *J* = 1.8 Hz, 1H), 7.29-7.26 (m, 1H), 7.14-7.10 (d, 2H, *J* = 8.8 Hz), 6.85-6.80 (d, 2H, *J* = 8.8 Hz), 6.63 (dd, 1H, *J* = 1.6 Hz and *J* = 3.2 Hz), 5.97 (s, 2H), 4.62 (s, 3H), 4.37 (t, 2H, *J* = 7.0 Hz), 2.62 (t, 2H, *J* = 7.0 Hz), 2.30-2.19 (m, 2H). ESI-HRMS calculated for $C_{22}H_{22}N_7O_4$ [M + H]$^+$, 448.1727; Calcd. 448.1733.

2-((1E,3E)-5-((E)-3-(6-((2-(2-(4-(3-(5-amino-2-(furan-2-yl)-7H-pyrazolo[4,3-e][1,2,4]triazolo[1,5-c]pyrimidin-7-yl)propyl)phenoxy)acetamido)ethyl)amino)-6-oxohexyl)-3-methyl-5-sulfonato-1-(3-sulfonatopropyl)indolin-2-ylidene)penta-1,3-dien-1-yl)-3,3-dimethyl-1-(3-sulfonatopropyl)-3H-indol-1-ium-5-sulfonate, triethylammonium salt **9**.

To a solution of **6a** (1 eq., 0.7 mg, 0.0015 mmol) in DMF (0.3 mL) was added Et_3N (1.1 eq., 0.0002 mL, 0.0016 mmol) and Alexa Fluor® 647 NHS Ester (0.54 eq., 1.0 mg, 0.0008 mmol). The flask was protected from light, and the mixture was stirred overnight. The crude product was then directly purified by HPLC (column A, H_2O/AN : from 100/0 to 70/30, 40 min, t_R = 27.8 min) to afford after lyophilization a blue solid (1.2 mg, 62 %). ^{1}H NMR (D_2O-d_4, δ ppm): 7.87-7.84 (m, 1H), 7.79-7.76 (m, 1H), 7.75-7.71 (m, 2H), 7.59-7.57 (m, 2H), 7.50-7.48 (m, 1H), 7.28 (d, 1H, *J* = 8.3 Hz), 7.06 (d, 1H, *J* = 3.3 Hz), 6.92 (d, 1H, *J* = 8.3 Hz), 6.64 (d, 2H, *J* = 8.7 Hz), 6.53 (dd, 1H, *J* = 1.6 Hz and *J* = 3.2 Hz), 6.29-6.19 (m, 4H), 5.87 (d, 1H, *J* = 13.7 Hz), 5.37 (s, 1H), 4.21-4.19 (m, 2H), 4.16-4.13 (m, 2H), 4.08-4.02 (q, NCH_2), 3.96 (d, 1H, *J* = 14.6 Hz), 3.86-3.82 (m, 2H), 3.76 (d, 1H, *J* = 14.6 Hz), 3.66-3.52 (m, 1H), 3.57-3.54 (m, 1H), 3.11-3.00 (m, 2H), 2.94-2.91 (m, 2H), 2.84 (t, 2H, *J* = 7.25 Hz), 2.55 (m, 2H), 2.22-2.11 (m, 2H), 2.08 (t, 2H, *J* = 7.25 Hz), 1.99-1.94 (m, 3H), 1.82 (s, 1H), 1.54 (s, 3H), 1.49 (s, 3H), 1.41 (s, 3H), 1.34-1.26 (m, 2H), 1.23-1.18 (m, 5H), 1.16 (t, NCH_3), 1.09-1.01 (m, 1H). ESI-HRMS calculated for $C_{59}H_{68}N_{11}O_{16}S_4$ [M + H]$^+$, 1314.3739; Calcd. 1314.3728. HPLC purity 99% (R_t = 14.0 min).

2-((1E,3E)-5-((E)-3-(6-((4-(2-(4-(3-(5-amino-2-(furan-2-yl)-7H-pyrazolo[4,3-e][1,2,4]triazolo[1,5-c]pyrimidin-7-yl)propyl)phenoxy)acetamido)butyl)amino)-6-oxohexyl)-3-methyl-5-sulfonato-1-(3-sulfonatopropyl)indolin-2-ylidene)penta-1,3-dien-1-yl)-3,3-dimethyl-1-(3-sulfonatopropyl)-3H-indol-1-ium-5-sulfonate, triethylammonium salt **10**.

To a solution of **6c** (1 eq., 0.741 mg, 0.0015 mmol) in DMF (0.3 mL) was added Alexa Fluor® 647 NHS Ester (0.543 eq., 1 mg, 0.0008 mmol) and Et_3N (1.1 eq., 0.0002 mL, 0.00162 mmol). The flask was protected from light, and the mixture was stirred overnight. The crude product was then directly purified by HPLC (column A, H_2O/AN : from 100/0 to 70/30, 40 min, t_R = 29.06 min) to afford after lyophilization a blue solid (1.5 mg, 76 %). ^{1}H NMR (MeOD-d_4, δ ppm): 7.83-7.65 (m, 5H), 7.56 (m, 2H), 7.48 (d, 1H, *J* = 8.4 Hz), 7.24 (d, 1H, *J* = 7.6 Hz), 6.98 (m, 1H), 6.91, (d, 1H, *J* = 7.6 Hz), 6.62 (d, 2H, *J* = 6.3 Hz), 6.51 (m, 1H), 6.23 (d, 2H, *J* = 8.8 Hz), 6.18-6.13 (m, 1H), 5.79 (d, 1H, *J* = 13.2 Hz), 4.10 (m, 4H), 3.94 (d, 1H, *J* = 14.4 Hz), 3.83-3.82 (m, 2H), 3.71 (d, 1H, *J* = 10.8 Hz), 3.10 (q, NCH_2), 3.01 (m, 2H), 2.95-2.80 (m, 8H), 2.49 (m, 2H), 2.14-2.11 (m, 6H), 1.99-1.92 (m, 4H), 1.49 (s, 3H), 1.41 (s, 3H), 1.37 (s, 3H), 1.23-1.20 (m, 6H), 1.17 (t, NCH_3), 1.06 (m, 2H). ESI-HRMS calculated for $C_{61}H_{72}N_{11}O_{16}S_4$ [M + H]$^+$, 1342.4052; Calcd. 1342.4041. HPLC purity 99% (R_t = 10.1 min).

(E)-*N*-(4-(2-(4-(3-(5-Amino-2-(furan-2-yl)-7H-pyrazolo[4,3-e][1,2,4]triazolo[1,5-c]pyrimidin-7-yl)propyl)phenoxy)acetamido)butyl)-6-(2-(4-(2-(5,5-difluoro-7-(thiophen-2-yl)-5H-4l4,5l4-dipyrrolo[1,2-c:2',1'-f][1,3,2]diazaborinin-3-yl)vinyl)phenoxy)acetamido)hexanamide **11**.

To a solution of **6c** (1 eq., 1.5 mg, 0.0029 mmol) in DMF (0.3 mL) was added Et_3N (3.0 eq., 1.2 µL, 0.0087 mmol) and BODIPY® 630/650-X NHS Ester (0.8 eq., 1.57 mg, 0.0024 mmol). The flask was protected from light, and the mixture was stirred overnight. The crude product was then directly purified by HPLC (column A, H_2O/AN : 50/50 to 0/100, 40 min, t_R = 29.9 min) to afford after lyophilization a blue solid (1.9 mg ; 61 %). ^1H NMR (MeOD-d_4, δ ppm): 8.05 (m, 2H), 7.74 (m, 1H), 7.60 (d, 1H, *J* = 7.6 Hz), 7.56 (d, 2H, *J* = 7.6 Hz), 7.46 (d, 1H, *J* = 3.9 Hz), 7.31 (s, 1H), 7.23 (d, 1H, *J* = 3.2 Hz), 7.18 (m, 2H), 7.00 (d, 1H, *J* = 4.4 Hz), 7.07-7.03 (m, 4H), 6.99 (d, 2H, *J* = 8.8 Hz), 6.65 (m, 1H), 4.36 (m, 2H), 4.29 (t, 2H, *J* = 6.8 Hz), 3.22 (t, 2H, *J* = 6.4 Hz), 3.12-3.10 (m, 2H), 2.56 (t, 2H, *J* = 6.4 Hz), 2.19-2.13 (m, 4H), 1.59-1.43 (m, 8H), 1.28-1.26 (m, 2H), 1.21-1.19 (m, 2H). ESI-HRMS calculated for $C_{54}H_{56}BF_2N_{12}O_6S$ $[M + H]^+$, 1049.4224; Calcd. 1049.4228. HPLC purity 96% (R_t = 13.6 min).

5-((4-(2-(4-(3-(5-Amino-2-(furan-2-yl)-7H-pyrazolo[4,3-e][1,2,4]triazolo[1,5-c]pyrimidin-7-yl)propyl)phenoxy)acetamido)butyl)carbamoyl)-2-(6-amino-3-imino-4,5-disulfo-3H-xanthen-9-yl)benzoic acid, triethylammonuim salt **12**

To a solution of **6c** (1 eq., 0.71 mg, 0.00141 mmol) in DMF (0.14 mL) was added Et_3N (1.1 eq., 0.0002 mL, 0.0016 mmol) and Alexa Fluor® 488 Carboxylic Acid, 2,3,5,6-Tetrafluorophenyl Ester, 5-isomer (0.8 eq., 1 mg, 0.0011 mmol). The flask was protected from light, and the mixture was stirred overnight. The crude product was then directly purified by HPLC (column B, H_2O/AN : 100/0 to 70/30, 20 min, t_R = 10.7 min) to afford after lyophilization an orange solid (0.47 mg ; 33 %). ^1H NMR (D_2O, δ ppm): 8.07 (s, 1H), 7.82 (s, 1H), 7.70 (m, 1H), 7.58 (s, 1H), 7.02 (d, 1H, *J* = 3.6 Hz), 6.85 (d, 2H, *J* = 9.6 Hz), 6.69-6.66 (m, 5H), 6.53 (m, 1H), 6.39 (d, 2H, *J* = 8.4 Hz), 4.09 (m, 4H), 3.24 (s, 2H), 3.10 (q, NCH_2), 3.07-3.05 (m, 2H), 2.27-2.25 (m, 2H), 2.03 (m, 2H), 1.39 (m, 4H), 1.17 (t, NCH_3). ESI-HRMS calculated for $C_{46}H_{40}N_{11}O_{13}S_2$ $[M + H]^+$, 1020.2411; Calcd. 1020.2405. HPLC purity 99% (R_t = 10.1 min).

4-((4-(3-(5-Amino-2-(furan-2-yl)-7H-pyrazolo[4,3-e][1,2,4]triazolo[1,5-c]pyrimidin-7-yl)propyl)phenoxy)methyl)benzenesulfonate ammonium salt **13**

To a solution of **7** (1 eq., 10 mg, 0.0266 mmol) in DMF (4.29 mL) under N_2 was added NaH (1 eq., 0.64 mg, 0.027 mmol) and the mixture was stirred for 15 min at room temperature before adding sodium 4-(bromomethyl)benzenesulfonate (1.1 eq., 8 mg, 0.0293 mmol). After 1 h stirring at room temperature, sodium 4-(bromomethyl)benzenesulfonate (1.1 eq., 8 mg, 0.0293 mmol) was added again, and the mixture was stirred for 45 min. The mixture was then treated with MeOH, concentrated *in vacuo* and purified by silica gel column chromatography (DCM/MeOH/NH_3: 85/15/1) to afford a white solid (6.3 mg, 42 %). ^1H NMR (MeOD-d_4, δ ppm) : 8.12 (s, 1H), 7.77-7.76 (m, 3H), 7.76 (m, 1H), 7.49 (d, 1H, *J* = 8.0 Hz), 7.45 (d, 2H, *J* = 8.4 Hz), 7.24 (dd, 1H, *J* = 0.4 Hz and *J* = 3.2 Hz), 7.04 (d, 2H, *J* = 8.8 Hz), 6.80 (d, 2H, *J* = 8.8 Hz), 6.67 (dd, 1H, *J* = 1.6 Hz and *J* = 3.2 Hz), 4.99 (s, 2H), 4.69 (br s, 1H), 4.38 (t, 2H, *J* = 7.0 Hz), 2.62 (t, 2H, *J* = 7.0 Hz), 2.27-2.24 (m, 2H). ESI HRMS calculated for $C_{26}H_{22}N_7O_5S^-$ $[M-H]^-$, 544.1401; Calcd. 544.1403. HPLC purity 96% (R_t = 10.8 min).

Pharmacological assays:

Cell culture for membrane binding assays and flow cytometry: HEK-293 cells stably expressing the $A_{2A}AR$ were grown in Dulbecco's Modified Eagle's Medium (DMEM) with 10% FBS, 100 units/ml penicillin, 100 mg/ml streptomycin, 2 mM L-glutamine and 0.500 mg/mL G418 Sulfate (Geneticin). Cells were maintained in a humidified atmosphere and sterile incubation conditions held at 37 ˚C and 5% CO_2 (g). A day prior to the experiment, cells were plated on a 96-well clear and flat bottom plate at 80-90% confluency in 100 µL of medium.

Radioligand binding assays: Cell membranes were prepared as reported.[1]

Binding assays were carried out using standard radioligands and membrane preparations from HEK-293 cells stably expressing the human (h) A_1, A_{2A} or A_3ARs or mouse (m) A_1, A_{2A} or A_3ARs. The radioligands used were: A_1AR, [^{3}H]8-cyclopentyl-1,3-dipropylxanthine **14**; A_{2A}AR, [^{3}H]**2**; A_3AR, [^{125}I]N^6-(4-amino-3-iodobenzyl)adenosine-5′-*N*-methyluronamide **16**. The radioligand for archival A_{2A}AR affinity data presented in Table 1 was [^{3}H]2-[p-(2-carboxyethyl)phenyl-ethylamino]-5′-*N*-ethylcarboxamidoadenosine **15**. Nonspecific binding was determined using 10 µM 8-[4-[[[[(2-aminoethyl)amino]carbonyl]methyl]oxy]phenyl]-1,3-dipropylxanthine **17** (A_1AR and A_{2A}AR) or 10 µM adenosine-5′-*N*-ethyluronamide **18** (A_3AR). HEK-293 cells expressing recombinant mA_1, A_{2A}, or A_3AR were used.
Protein was determined as reported.[2] In all the binding experiments, IC_{50} values and K_i values were calculated using GraphPad Prism software (San Diego, CA). Values are expressed as mean±SEM.

Fluorescent binding studies: All binding studies were done in triplicate. For saturation binding studies, cells were treated with 50 µL of **11** (MRS 7396) or **12** (MRS 7416), to achieve a final concentration from 0.19 to 400 nM, and 50 µL of Tris-HCl buffer containing 10 mM $MgCl_2$. Non-specific binding was determined with SCH442416 **3** (final concentration of 10 µM, in Tris-HCl buffer). For displacement experiments, cells were incubated simultaneously with 50 µL of 40 nM **11** or **12** (final concentration 10 nM) and 50 µL of the non-labeled displacing ligand at increasing concentrations. The total binding was measured in the absence of a displacing ligand, and non-specific binding was determined with 10 µM **3**. After 1 h at 37 °C (for both the saturation and displacement experiments), the medium was removed and the cells were carefully washed two times with 150 µL of ice-cold PBS (not containing Mg^{+2} or Ca^{+2}). The cells were treated with 40 µL of Corning Cellstripper (Mediatech, Manassas, VA) per well and then incubated at 37 °C for 10 min. To each well was subsequently added 160 µL of PBS (not containing Mg^{+2} or Ca^{+2}), and the cell fluorescence was analyzed with a BD FACSCalibur flow cytometer (Becton, Dickinson and Co., Franklin Lakes, NJ) with excitation at 635 nm (red diode laser, for **11**) or 488 nm (blue laser, for **12**) in conjunction with the software from BD Bioscience PlateManager and CellQuest. Data anlysis was performed with the Prism 5 (GraphPad, San Diego, CA) software.

1. Tosh, D.K., Paoletta, S., Chen, Z., Crane, S., Lloyd, J., Gao, Z.G., Gizewski, E.T., Auchampach, J.A., Salvemini, D., Jacobson, K.A. Structure-based design, synthesis by click chemistry and in vivo activity of highly selective A_3 adenosine receptor agonists. Med. Chem. Comm., 2015, 6:555-563.

2. Bradford, M. M. A rapid and sensitive method for the quantitation of microgram quantities of protein utilizing the principle of protein-dye binding. *Anal. Biochem.* **1976**, *72*, 248-254.

Molecular Modeling Methods

Protein preparation. The high-resolution $hA_{2A}AR$ X-ray structure in complex with the triazolo-triazine antagonist ZM241385, structurally related to the reference compound **3**, was retrieved from the Protein Data Bank (PDB)[1] (ID: 4EIY). Hydrogen atoms were added using the Protein Preparation Wizard tool implemented in the Schrödinger suite[2]. During the protein preparation, co-crystallized hetero groups and the fusion partner (BRIL) were removed. The protonation states of titrable residues were determined according to H-bond patterns with surrounding residues. To this aim, all water molecules present in the X-Ray construct were retained during the protein preparation procedure. However, for the subsequent docking analysis only water molecules in the first solvation sphere of the ligand were kept. According to H-bond pattern analysis His75/278/306 and His155/230/250 were protonated on the N^{δ} and the N^{ε}, respectively, whereas His264 (establishing a salt bridge with Glu169) was considered doubly protonated. The native sequence of the $hA_{2A}AR$ as well as missing side chains of residues whose backbone coordinates were observed in the X-ray structure were restored by building a homology model with Prime[3].

Docking. Structures of selected ligands were built and prepared for docking using the Builder and the LigPrep tools implemented in the Schrödinger suite[4]. The structures were minimized using the OPLS_2005 force field. Molecular docking was performed with the Glide package from the Schrödinger suite[5], with the barycenter of the co-crystallized ligand representing the center of the Glide Grid (inner box: 14 x 14 x 14 Å; outer box extended by 20 Å in each direction from the inner box). Docking was performed considering the protein binding sites residues rigid by using the standard precision (SP) scoring function. Ligands were docked at the $hA_{2A}AR$ by retaining a variable number (depending upon the specific ligand considered) of non-overlapping water molecules according to the following protocol: ligands were first docked at the $hA_{2A}AR$ structure without water molecules; the best docking poses so obtained were superimposed with the $hA_{2A}AR$ structure containing water molecules in the first solvation sphere of the co-crystallized ligand; after the superimposition, non-overlapping water molecules were identified; ligands were therefore redocked at the $hA_{2A}AR$ containing those water molecules. In a few cases, iterative cycles of removal of non-overlapping water molecules and ligand docking were performed until the SP score did not further improve.

Molecular Dynamics. MD system setup, equilibration, and production were performed with the HTMD[6] module (Acellera, Barcelona Spain, version 1.5.4). The ligand-protein complexes were embedded into an 80 x 80 Å 1-palmitoyl-2-oleoyl-sn-glycero-3-phosphocholine (POPC) membrane leaflet generated through the VMD Membrane Plugin tool[7]. Overlapping lipids (within 0.6 Å) were removed upon protein insertion and the systems were solvated with TIP3P[8] water and neutralized by Na^+/Cl^- counter-ions (final concentration 0.154 M). MD simulations with periodic boundaries conditions were carried out with the ACEMD engine (Acellera, version 2016.10.27)[9] using the CHARMM36[10,11]/CGenFF(3.0.1)[12,13] force fields for lipid and protein, and ligand atoms, respectively. Ligand parameters were retrieved from the ParamChem service (https://cgenff.paramchem.org, accessed 04/2017, version 1.0.0) with no further optimization. After initial validation, the atom types for compounds **12** were manually assigned to enforce the equivalency of the atoms on the two terminal aryl rings of the fluorophore moiety, consistently with previous MD studies performed on AlexaFluor488[14,15]. As for the specific purpose of this study atomic charges on the so-defined atom types were not optimized, the electrostatic contribution to the total ligand-protein interaction energy for this ligand was evaluated only qualitatively and will not be described in detail. The systems were equilibrated through a 5000-step minimization followed by 40 ns of MD simulation in the NPT ensemble by applying initial constrains (0.8 for the ligand atoms, 0.85 for alpha carbon atoms, and 0.4 for the other protein atoms) that were linearly reduced after 20 ns. During the equilibration procedure, the temperature was maintained at 310 K using a Langevin thermostat with a low damping constant of 1 ps^{-1}, and the pressure was maintained at 1 atm using a Berendensen barostat. Bond

lengths involving hydrogen atoms were constrained using the M-SHAKE[16] algorithm. The equilibrated systems were subjected to 30 ns of unrestrained MD simulations run in triplicate for each ligand-protein complex (NVT ensemble, timestep = 2 fs, damping constant = 0.1 ps^{-1}). Long-range Coulomb interactions were handled using the particle mesh Ewald summation method (PME)[17] with grid size rounded to the approximate integer value of cell wall dimensions. A non-bonded cutoff distance of 9 Å with a switching distance of 7.5 Å was used. All simulations were run on three NVIDIA GeForce GTX (970, 980Ti, and 1080).

MD Trajectory Analysis. MD trajectory analysis was performed with an in-house script exploiting the NAMD 2.10[18] *mdenergy* function and the RMSD trajectory tool (RSMDTT) implemented in VMD[7]. All simulations were run in triplicate and selection of representative trajectories and of lowest interaction energy (IE) ligand-protein complexes were based upon the total ligand–protein interaction energy (IE_{tot}) expressed as the sum of van der Waals (IE_{vdW}) and electrostatic (IE_{ele}) contribution as previously described[19]. IE vs simulation time graph was generated with an in-house script exploiting Gnuplot[20].

Modeling References

1. Bernstein, F. C.; Koetzle, T. F.; Williams, G. J.; Meyer Jr., E. E.; Brice, M. D.; Rodgers, J. R.; Kennard, O.; Shimanouchi, T.; Tasumi, M. The Protein Data Bank: A computer-based archival file for macromolecular structures. *J. Mol. Biol.* **1977**, *112*, 535–542.

2. Sastry, G. M.; Adzhigirey, M.; Day, T.; Annabhimoju, R.; Sherman, W. Protein and ligand preparation: parameters, protocols, and influence on virtual screening enrichments *J. Comput. Aided Mol. Des.* **2013**, *27*, 221–234.

3. *Prime*, Schrödinger, LLC, New York, NY, 2017.

4. *LigPrep*, Schrödinger, LLC, New York, NY, 2017.

5. *Glide*, Schrödinger, LLC, New York, NY, 2017.

6. Doerr, S.; Harvey, M. J.; Noé, F.; De Fabritiis, G. HTMD: High-Throughput Molecular Dynamics for Molecular Discovery. *J. Chem. Theory Comput.*, **2016**, *12*, 1845-1852.

7. Humphrey, W.; Dalke, A.; Schulten, K. VMD - Visual molecular dynamics. *J. Mol. Graphics*, **1996**, *14*, 33–38.

8. Jorgensen W. L.; Chandrasekhar, J.; Madura, J. D.; Impey, R. W.; Klein, M. L. Comparison of simple potential functions for simulating liquid water. *J. Chem. Phys.* **1983**, *79*, 926–935.

9. Harvey, M.; Giupponi, G.; De Fabritiis, G. ACEMD: Accelerated molecular dynamics simulations in the microseconds timescale. *J. Chem. Theory Comput.* **2009**, *5*, 1632–1639.

10. Best, R. B.; Zhu, X.; Shim, J.; Lopes, P. E. M.; Mittal, J.; Feig, M.; MacKerell Jr., A. D. Optimization of the additive CHARMM all-atom protein force field targeting improved sampling of the backbone phi, psi and side-chain chi1 and chi2 dihedral angles. *J. Chem. Theory Comput.* **2012**, *8*, 3257–3273.

11. Klauda, J. B.; Venable, R. M.; Freites, J. A.; O'Connor, J. W.; Tobias, D. J.; Mondragon-Ramirez, C.; Vorobyov, I.; MacKerell Jr., A. D.; Pastor, R. W. Update of the CHARMM all-atom additive force field for lipids: validation on six lipid types. *J. Phys. Chem. B*, **2010**, *114*, 7830-7843.

12. Vanommeslaeghe, K.; MacKerell, A. D., Jr., Automation of the CHARMM General Force Field (CGenFF) I: bond perception and atom typing. *J. Chem. Inf. Model.* **2012,** *52*, 3144-3154.

13. Vanommeslaeghe, K.; Raman, E. P.; MacKerell, A. D., Jr., Automation of the CHARMM General Force Field (CGenFF) II: assignment of bonded parameters and partial atomic charges. *J. Chem. Inf. Model.* **2012,** *52*, 3155-3168.

14. Kräutler, V.; Van Gunsteren, W. F.; Hünenberger, P. H. A Fast SHAKE algorithm to solve distance constraint equations for small molecules in molecular dynamics simulations. *J. Comput. Chem.* **2001**, *22*, 501-508.

15. Corry, B.; Jayatilaka, D. Simulation of Structure, orientation and Energy transfer between AlexaFluor Molecules Attached to MscL. *Bioph. J.* **2008**, *95*, 2711-2721.

16. Walczewska-Szewc, K.; Deplaxes, E.; Corry, B. Comparing the Ability of Enhanced Sampling Molecular Dynamics Methods To Reproduce the Behavior of Fluorescent Labels on Proteins. *J. Chem. Theory Comput.* **2015**, *11*, 3455-3465.

17. Essmann, U.; Perera, L.; Berkowitz, M. L.; Darden, T.; LeeH.; Pedersen L. G. A. Smooth particle mesh Ewald method. *J. Chem. Phys.* **1995**, *103*, 8577-8593.

18. Phillips, J. C.; Braun, R.; Wang, W.; Gumbart, J.; Tajkhorshid, E.; Villa, E.; Chipot, C.; Skeel, R. D.; Kale, L.; Schulten, K. Scalable molecular dynamics with NAMD *J. Comput. Chem.* **2005**, *26*, 1781-1802.

19. Williams, T.; Kelley, C. Gnuplot 5.0: An Interactive Plotting Program, version 5.0.3, 2017; http://gnuplot.info (accessed April 10, **2017**).

20. Toti, K. S.; Osborne, D.; Ciancetta, A.; Boison, D.; Jacobson, K. A. South (S)- and North (N)-Methanocarba-7-Deazaadenosine Analogues as Inhibitors of Human Adenosine Kinase. *J. Med. Chem.* **2016**, 59, 6860-6877.

Molecular Modeling Results: Tables and Figures

Table S1. Parameters considered for the selection of a representative trajectory among three replicas: protein alpha carbon atoms (Cα) average RMSD, ligand average RMSD, and slope of the dynamic scoring function (DSFslope). RMSD values are in Å and DSF is adimensional. Selected runs are marked in bold.

Ligand	Run Number	DSFslope [adimensional]	Average RMSD [Å]	
			Ligand	Calpha
**2**	1	-38.325	1.819	1.611
	2	-14.632	3.892	1.970
	**3**	**-59.813**	**1.524**	**1.871**
**6c** (BM1)	1	-23.526	3.587	1.657
	**2**	**-23.990**	**4.231**	**1.575**
	3	-21.610	4.045	1.468
**6c** (BM2)	1	-26.236	2.866	1.478
	2	-22.169	3.545	1.767
	**3**	**-34.341**	**2.368**	**1.567**
**12** (BM1)	1	-12.775	5.465	1.560
	2	-28.363	3.106	1.676
	**3**	**-30.760**	**3.083**	**1.546**
**12** (BM2)	1	-71.977	3.268	1.505
	**2**	**-130.815**	**1.835**	**1.420**
	3	-86.127	3.004	1.409
**13** (BM1)	1	-10.573	6.704	1.652
	**2**	**-27.083**	**3.129**	**1.749**
	3	-19.874	3.289	1.789
**13** (BM2)	1	-26.010	3.355	1.695
	2	-22.557	5.427	1.368
	**3**	**-28.201**	**2.256**	**1.511**
**13** (BM3)	1	-21.335	5.235	1.494
	**2**	**-26.411**	**4.350**	**1.458**
	3	-17.947	4.426	1.832

Figure S1. Most energetically favored ligand-protein structure (Interaction Energy = -89.544 kcal/mol)) obtained for **2**-$hA_{2A}AR$ complex in the selected MD run starting from the docking pose. In this snapshot the ligand features the same interaction pattern observed for the initial docking pose, thus validating the quality of the ligand-protein interaction predicted by docking. Side view facing TM6, TM7, and TM1 (from the left).

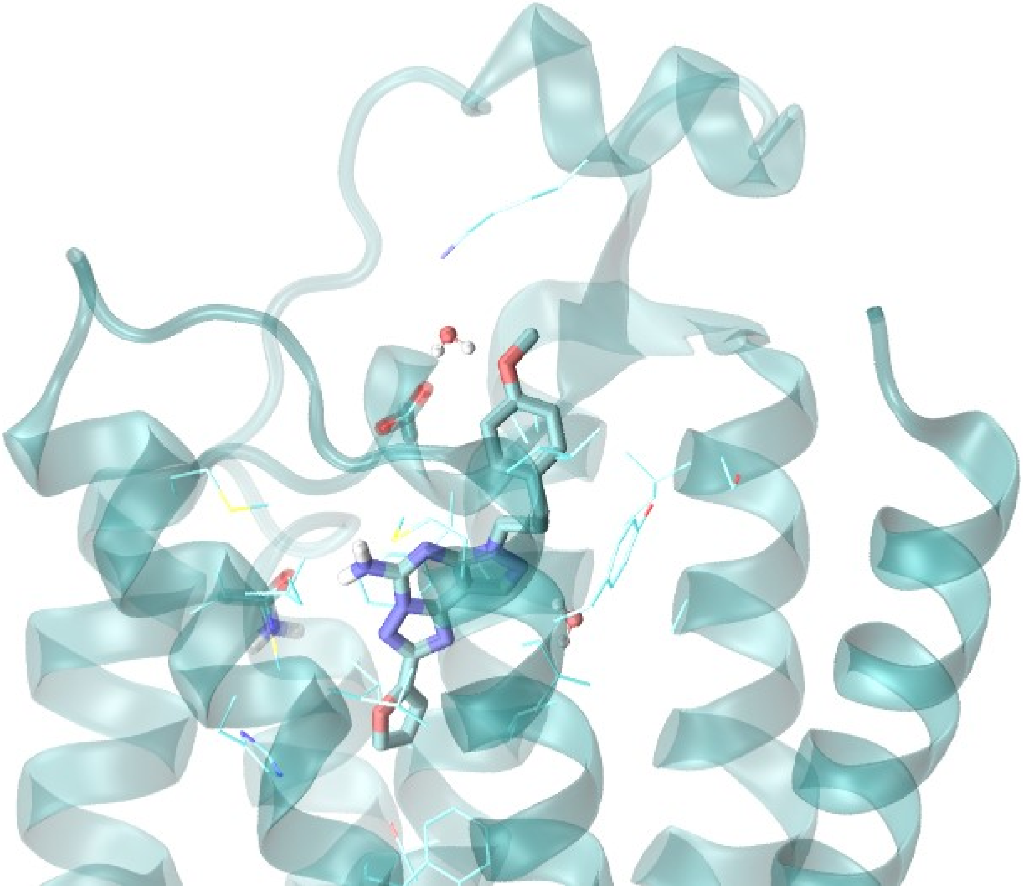

Figure S2. Two alternative binding modes obtained for compound **6c**, the synthetic precursor of **11**, at the $hA_{2A}AR$. In the most energetically favored docking complex (orange carbon sticks, docking score =-12.077 kcal/mol) the points toward TM4 and TM5, the amide moiety establishes a H-bond with the sidechain of E169, and the terminal amine group engages in H-bond interactions with the backbone of E169 (EL2) and the sidechain of K150 (EL2). In the alternative binding mode (green carbon sticks, docking score =-10.994 kcal/mol), the tail points toward TM1 and TM2 and does not establish additional interactions. Residues establishing polar (dashed orange lines) and π-π interactions with the docked ligands are represented as thin sticks. Non-polar hydrogen atoms are omitted.

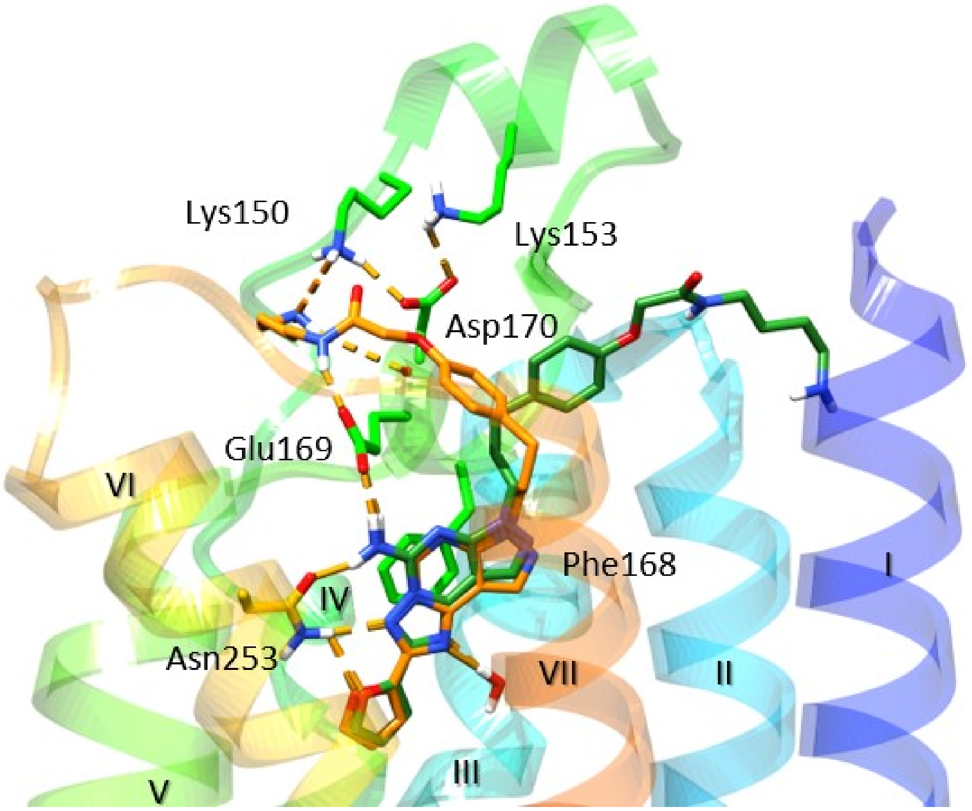

Figure S3. Three-dimensional representation (A) and schematic depiction (B) of the distance between the terminal ammine group and the centroids of the aromatic moieties in the fluorophore group of **11**. (C) Most energetically favored ligand-protein complexes obtained after MD simulation, starting from **6c**-$hA_{2A}AR$ docked complexes. Aromatic (solid surface) and hydrophobic (wireframe surface) regions in the proximity of the terminal amine moiety are colored according to the distance from the nitrogen atom as follows: 5 Å = magenta, 13 Å = green, and 14 Å = yellow. As depicted, only in one orientation the proximity of aromatic/hydrophobic regions in the protein (colored arrow) are compatible with the placement of the aromatic moieties of the fluorophore group of **11**. Side view, facing TM6, TM7, and TM1 (from the left).

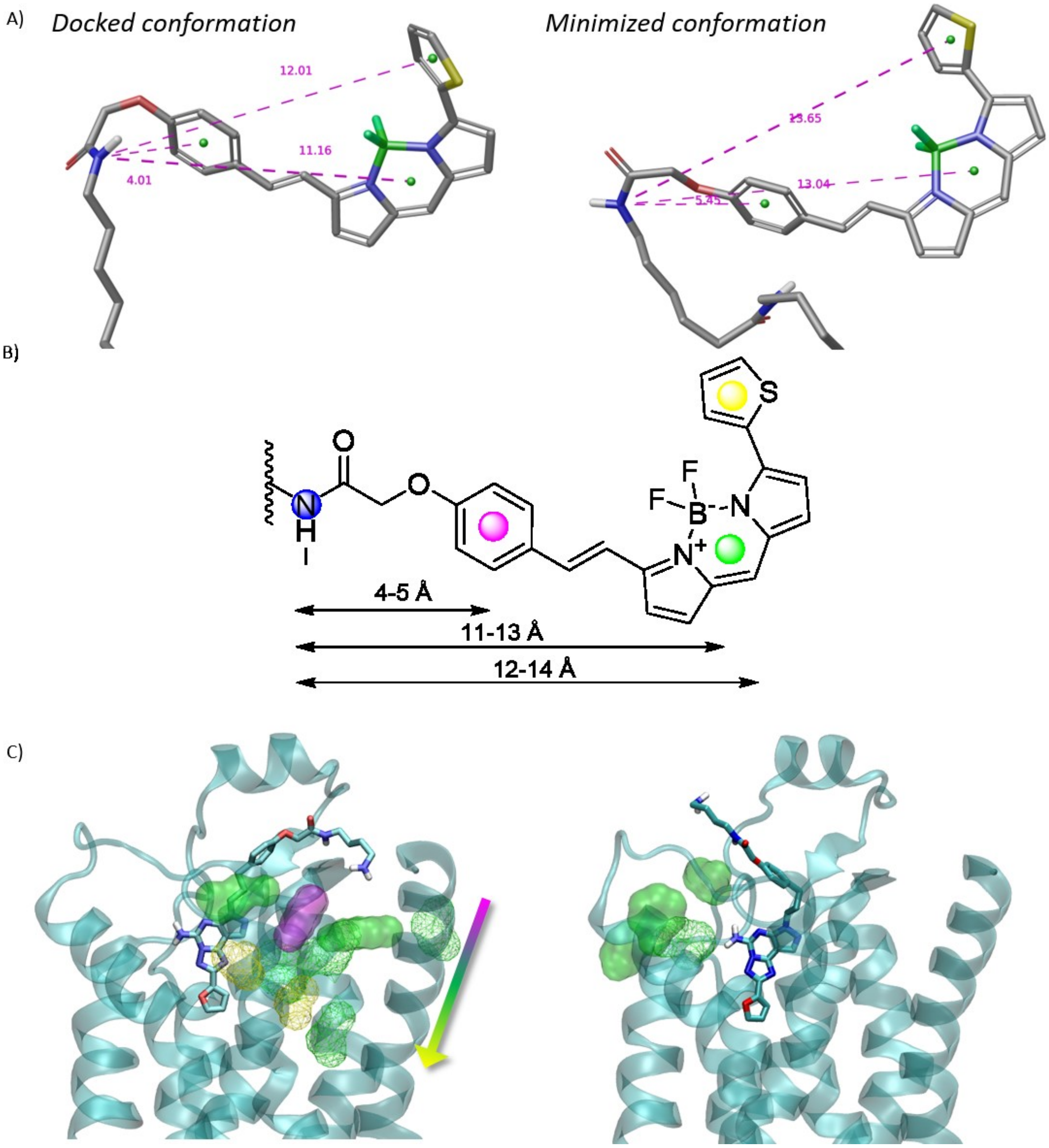

Figure S4. Superimposition of the most energetically favorable **12**-hA_{2A}AR complexes obtained after three MD simulation starting from BM2: the three replicas converged in a unique binding mode. The structures are colored according to the IE value, the lower (more favorable) the value the darker the color.

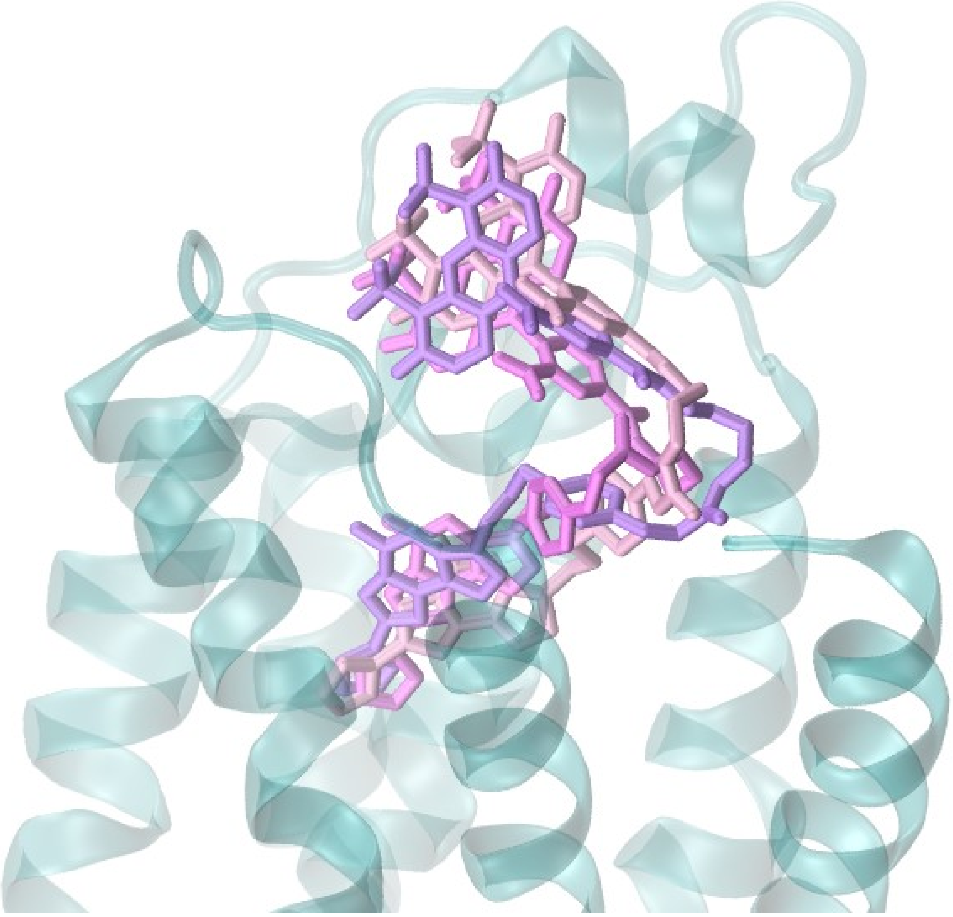

Figure S5. (A) Superimposition of the most energetically favorable **13**-hA_{2A}AR complexes obtained after MD simulation starting from three different binding modes (BM1 = cyan, BM2 = magenta, BM3 = orange): the three different initial poses converged in a unique binding mode. (B) Ligand-protein complex with the lowest interaction energy (IE = -201.590 kcal/mol) obtained after MD simulation starting from BM3: with respect to its initial conformation, the 7-phenylpropyl ring of the ligand moves toward TM7 and establishes a π-π stacking interaction with Y271 (7.36). Both A and B are a side view facing TM6, TM7, and TM1 (from the left).

A) B)

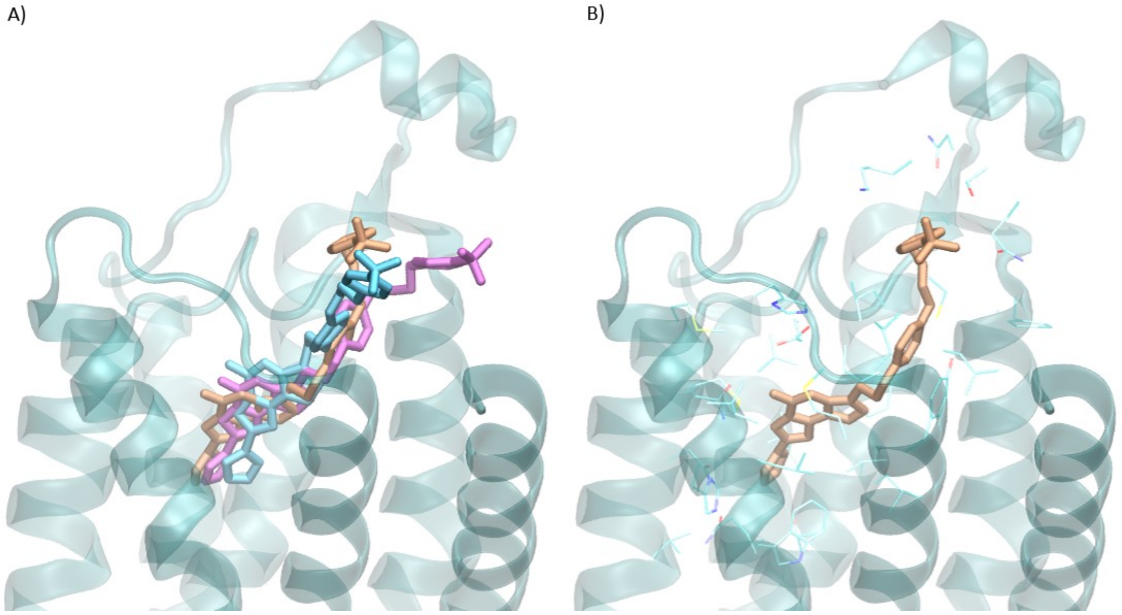

Pharmacological Results

Inhibition of whole cell binding of fluorescent probe 12 by agonists:

Although the inhibition of binding of AlexaFluor488 conjugate **12** provided the expected affinities when employing antagonists, the inhibition by agonists was complex, possibly due to multiple affinity states of this GPCR for agonists. Further study is required.

Agonists 2-[p-(2-carboxyethyl)phenyl-ethylamino]-5′-*N*-ethylcarboxamidoadenosine **15** and 6-(2,2-diphenylethylamino)-9-((2*R*,3*R*,4*S*,5*S*)-5-(ethylcarbamoyl)-3,4-dihydroxytetrahydrofuran-2-yl)-*N*-(2-(3-(1-(pyridin-2-yl)piperidin-4-yl)ureido)ethyl)-9*H*-purine-2-carboxamide **19** bound with K_i values, respectively, of 4.0 and 99 nM.

Demonstration of antagonist action at the $hA_{2A}AR$:

Figure S6. Right shifts of the $hA_{2A}AR$ curve for activation, i.e. cyclic AMP accumulation, by CGS21680 **15**, induced by antagonists **11** (A) and **13** (B). Results are expressed and mean±SEM from 2-3 experiments performed in duplicate. The EC_{50} of CGS21680 **15** alone was 0.89±0.17 nM, in the presence of **11** (1000 nM), EC_{50} = 128±35 nM; in the presence of **13** (100 nM), EC_{50} = 10.2±2.3 nM.

A

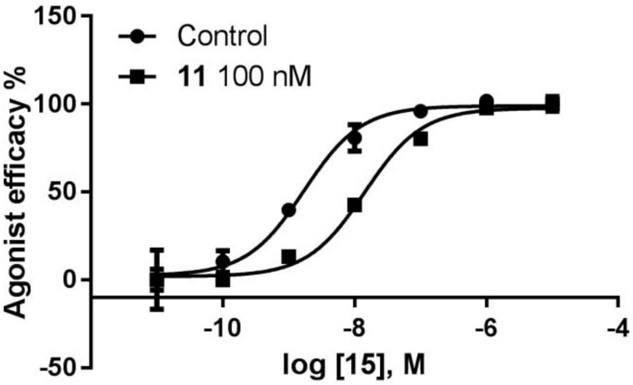

B

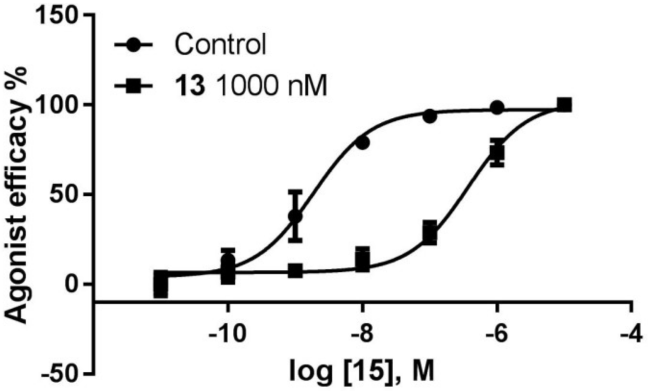

Chinese hamster ovary (CHO) cells stably expressing the human $A_{2A}AR$ were cultured in Dulbecco's Modified Eagle Medium (Mediatech, Manassas, VA) supplemented with 10% fetal bovine serum, 100 units/ml penicillin, 100 µg/ml streptomycin and 2 µmol/ml glutamine. Cells were plated in 96-well plates in 100 µl medium. After 24 h, the medium was removed and cells were washed three times with 100 µl DMEM, containing 50 mM HEPES, pH 7.4. Cells were treated with antagonists (or medium for control) in the presence of rolipram (10 µM) and adenosine deaminase (3 units/ml) and 5 min later with agonist for 20 min. The reaction was terminated by removal of the supernatant, and cells were lysed upon the addition of 100 µl of lysis buffer (0.3% Tween-20). cAMP was measured using ALPHAScreen cAMP kits (PerkinElmer, Boston, MA) as instructed by the manufacturer.

Off-target interactions for selected compounds (K_i in radioligand binding inhibition <10 µM)
Refer to: http://pdspdb.unc.edu for full list of comprehensive screen at 45 targets.

PDSP 46674, MRS7354 (**6c**)
$5HT_{2A}$ serotonin receptor:

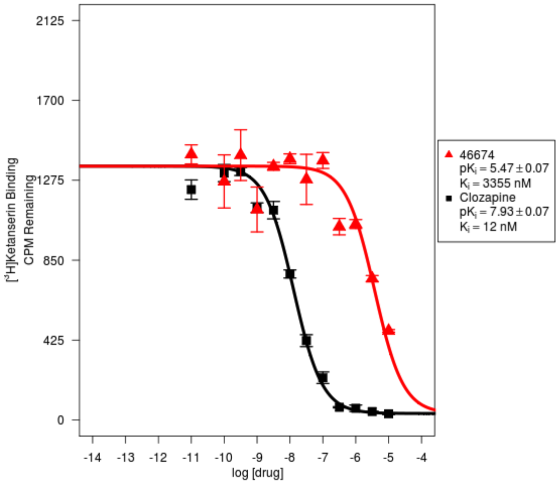

$5HT_{2B}$ serotonin receptor:

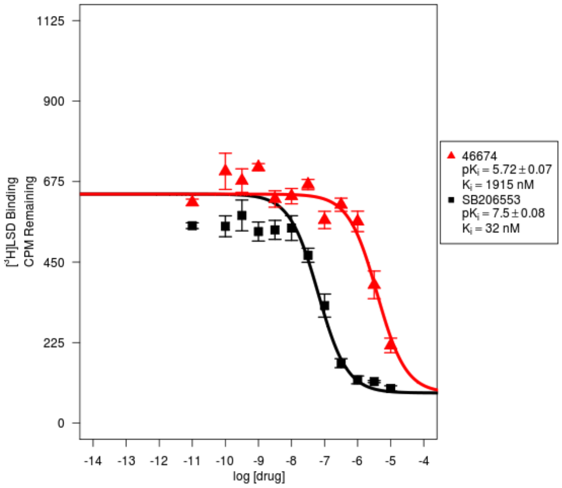

PDSP 48400, MRS7352 (**13**)

None detected.

6b, MRS7353

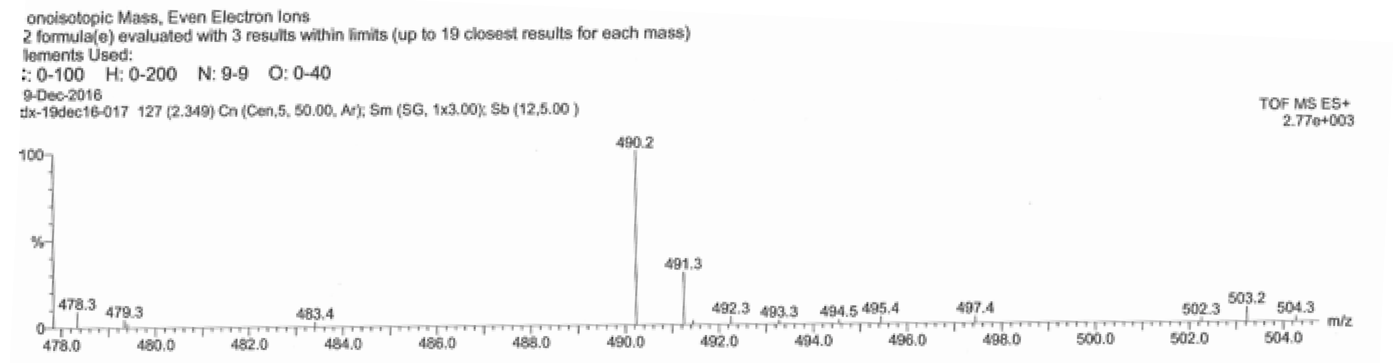

Minimum:				-2.0		
Maximum:		10.0	10.0	1000.0		
Mass	Calc. Mass	mDa	PPM	DBE	i-FIT	Formula
490.2321	490.2315	0.6	1.2	15.5	5.4	C24 H28 N9 O3
	490.2374	-5.3	-10.8	6.5	20.9	C17 H32 N9 O8
	490.2221	10.0	20.4	2.5	63.0	C13 H32 N9 O11

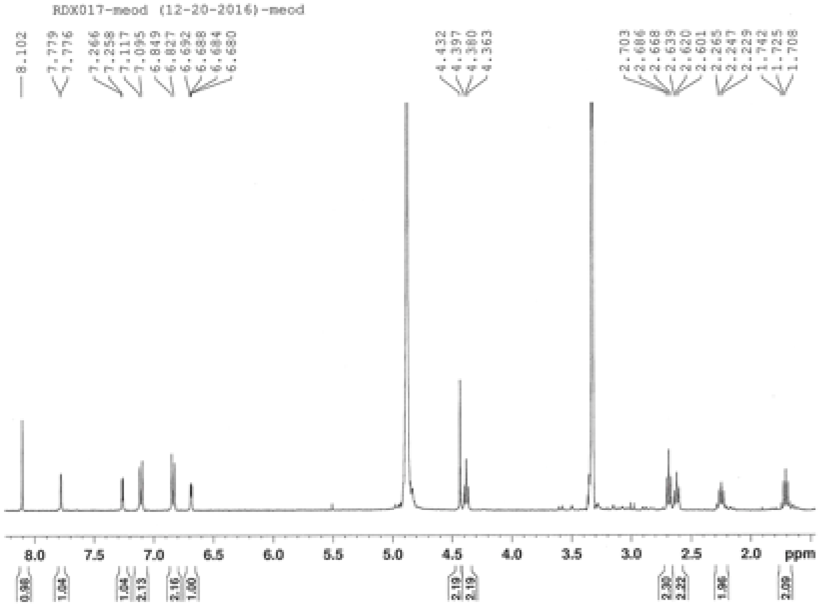

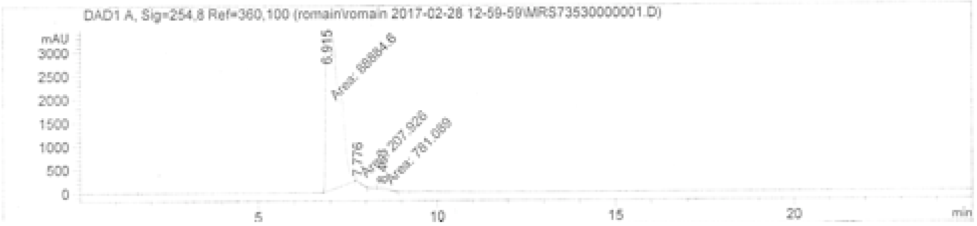

Signal 1: DAD1 A, Sig=254,8 Ref=360,100

Peak #	RetTime [min]	Type	Width [min]	Area [mAU*s]	Height [mAU]	Area %
1	6.915	MM	0.4503	8.88846e4	3289.68872	98.8995
2	7.776	MM	0.0852	207.92612	40.68391	0.2314
3	8.463	MM	0.3377	781.08868	38.54378	0.8691
Totals :				8.98736e4	3368.91641	

6c, MRS7354

Monoisotopic Mass, Even Electron Ions
93 formula(e) evaluated with 3 results within limits (up to 19 closest results for each mass)
Elements Used:
C: 0-100 H: 0-200 N: 9-9 O: 0-40
21-Dec-2016
rdx-21dec16-018 184 (3.402) Cn (Cen,5, 50.00, Ar); Sm (SG, 1x3.00); Sb (12,5.00)

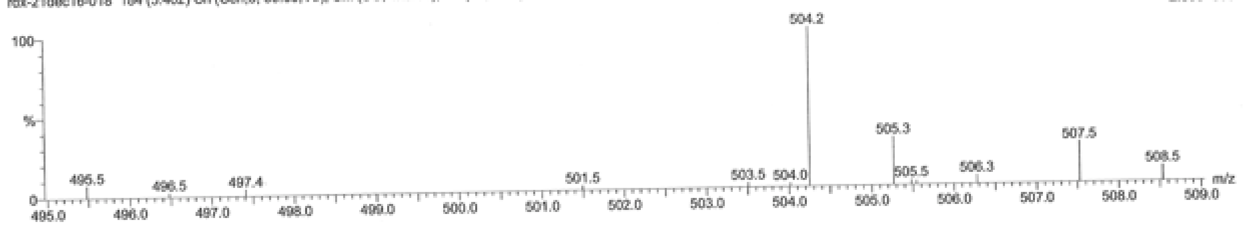

Minimum:				-2.0		
Maximum:		10.0	10.0	1000.0		
Mass	Calc. Mass	mDa	PPM	DBE	i-FIT	Formula
504.2476	504.2472	0.4	0.8	15.5	4.4	C25 H30 N9 O3
	504.2530	-5.4	-10.7	6.5	18.8	C18 H34 N9 O8
	504.2378	9.8	19.4	2.5	59.2	C14 H34 N9 O11

RDX018-2-meod (12-23-2016)-meod

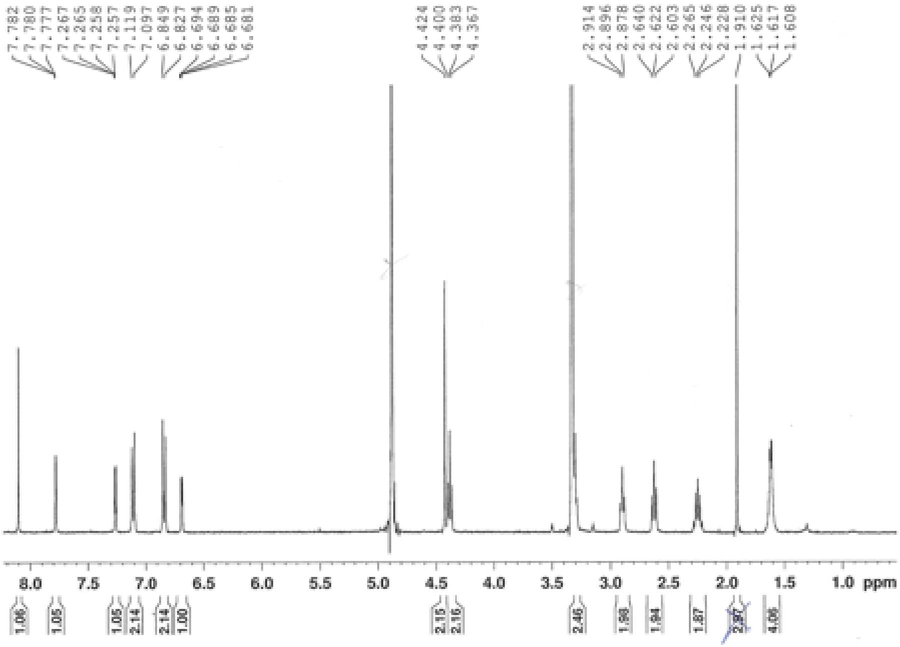

6d, MRS7355

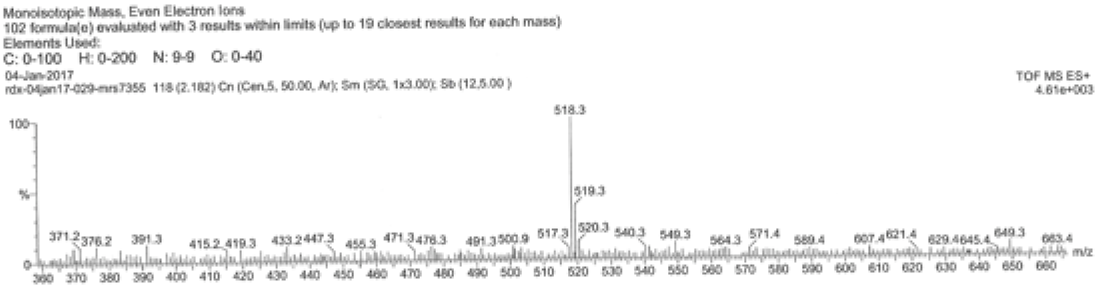

Minimum:				-2.0		
Maximum:		10.0	10.0	1000.0		
Mass	Calc. Mass	mDa	PPM	DBE	i-FIT	Formula
518.2631	518.2628	0.3	0.6	15.5	81.1	C26 H32 N9 O3
	518.2687	-5.6	-10.8	6.5	174.2	C19 H36 N9 O8
	518.2534	9.7	18.7	2.5	268.5	C15 H36 N9 O11

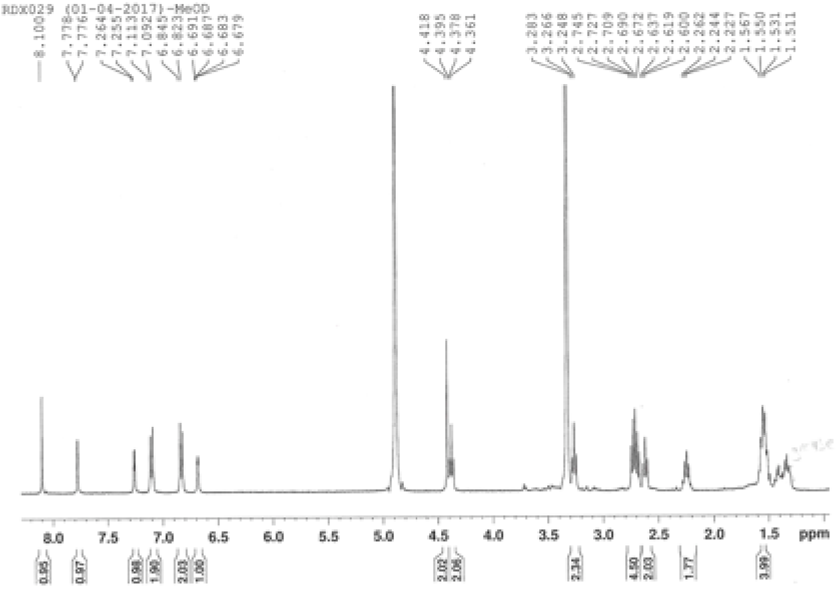

6e, MRS7356

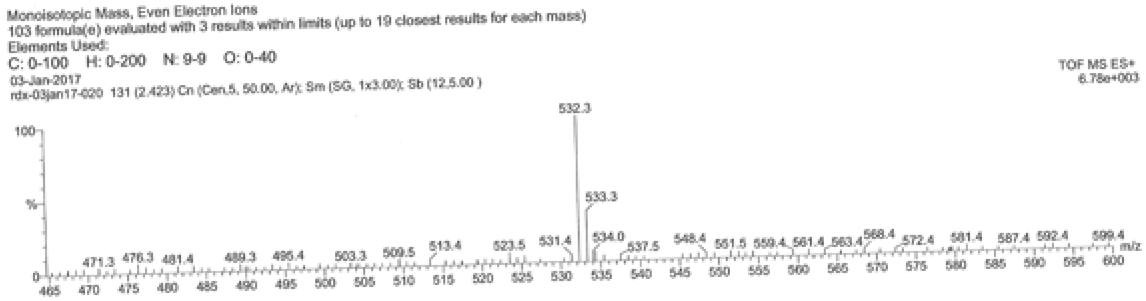

Minimum:				-2.0		
Maximum:		10.0	10.0	1000.0		
Mass	Calc. Mass	mDa	PPM	DBE	i-FIT	Formula
532.2776	532.2785	-0.9	-1.7	15.5	6.6	C27 H34 N9 O3
	532.2843	-6.7	-12.6	6.5	62.2	C20 H38 N9 O8
	532.2691	8.5	16.0	2.5	160.6	C16 H38 N9 O11

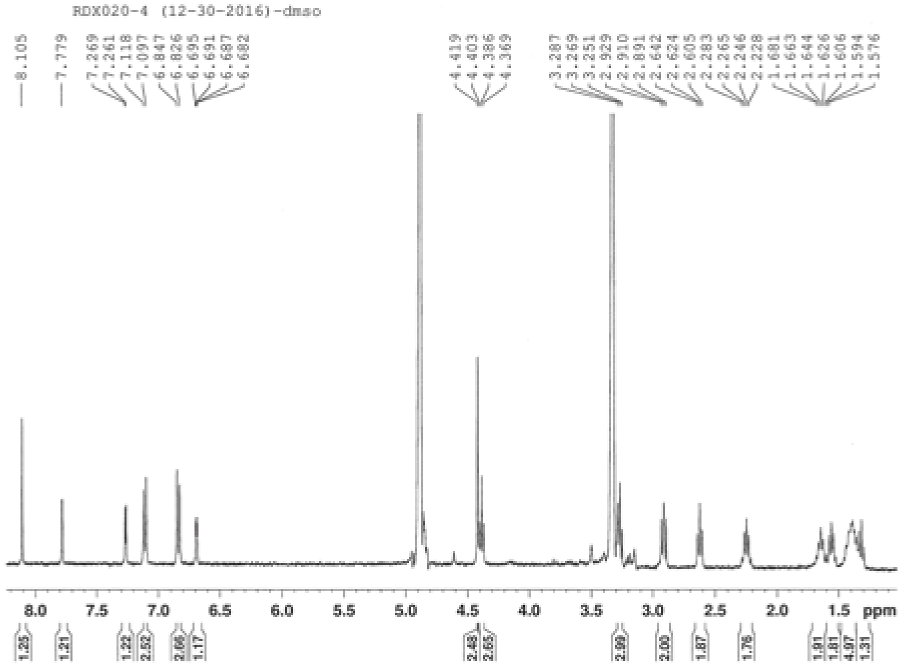

9, MRS7322

ngle Mass Analysis
lerance = 10.0 mDa / DBE: min = -2.0, max = 1000.0
ement prediction: Off
umber of isotope peaks used for i-FIT = 3

onoisotopic Mass, Even Electron Ions
28 formula(e) evaluated with 7 results within limits (up to 19 closest results for each mass)
lements Used:
: 0-100 H: 0-200 N: 11-11 O: 0-40 32S: 4-4
2-Dec-2016
RDX-22dec16-022 53 (0.980) Cn (Cen,5, 50.00, Ar); Sm (SG, 1x3.00); Sb (12,5.00); Sb (15,10.00); Sm (SG, 3x5.00)

TOF MS ES-
3.49e+002

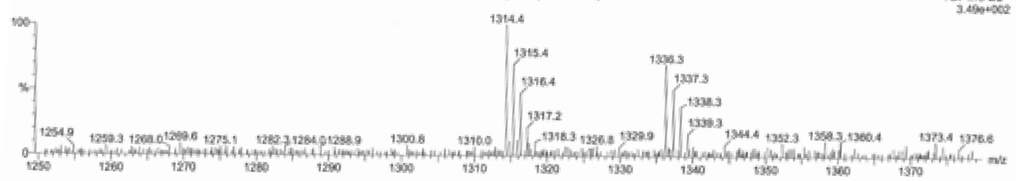

Mass	Calc. Mass	mDa	PPM	DBE	i-FIT	Formula
Minimum:				-2.0		
Maximum:		10.0	10.0	1000.0		
1314.3739	1314.3728	1.1	0.8	31.5	13.0	C59 H68 N11 O16 32S4
	1314.3752	-1.3	-1.0	0.5	41.9	C34 H80 N11 O34 32S4
	1314.3763	-2.4	-1.8	53.5	9.7	C77 H60 N11 O3 32S4
	1314.3693	4.6	3.5	9.5	30.3	C41 H76 N11 O29 32S4
	1314.3787	-4.8	-3.7	22.5	18.0	C52 H72 N11 O21 32S4
	1314.3670	6.9	5.2	40.5	10.2	C66 H64 N11 O11 32S4
	1314.3822	-8.3	-6.3	44.5	9.9	C70 H64 N11 O8 32S4

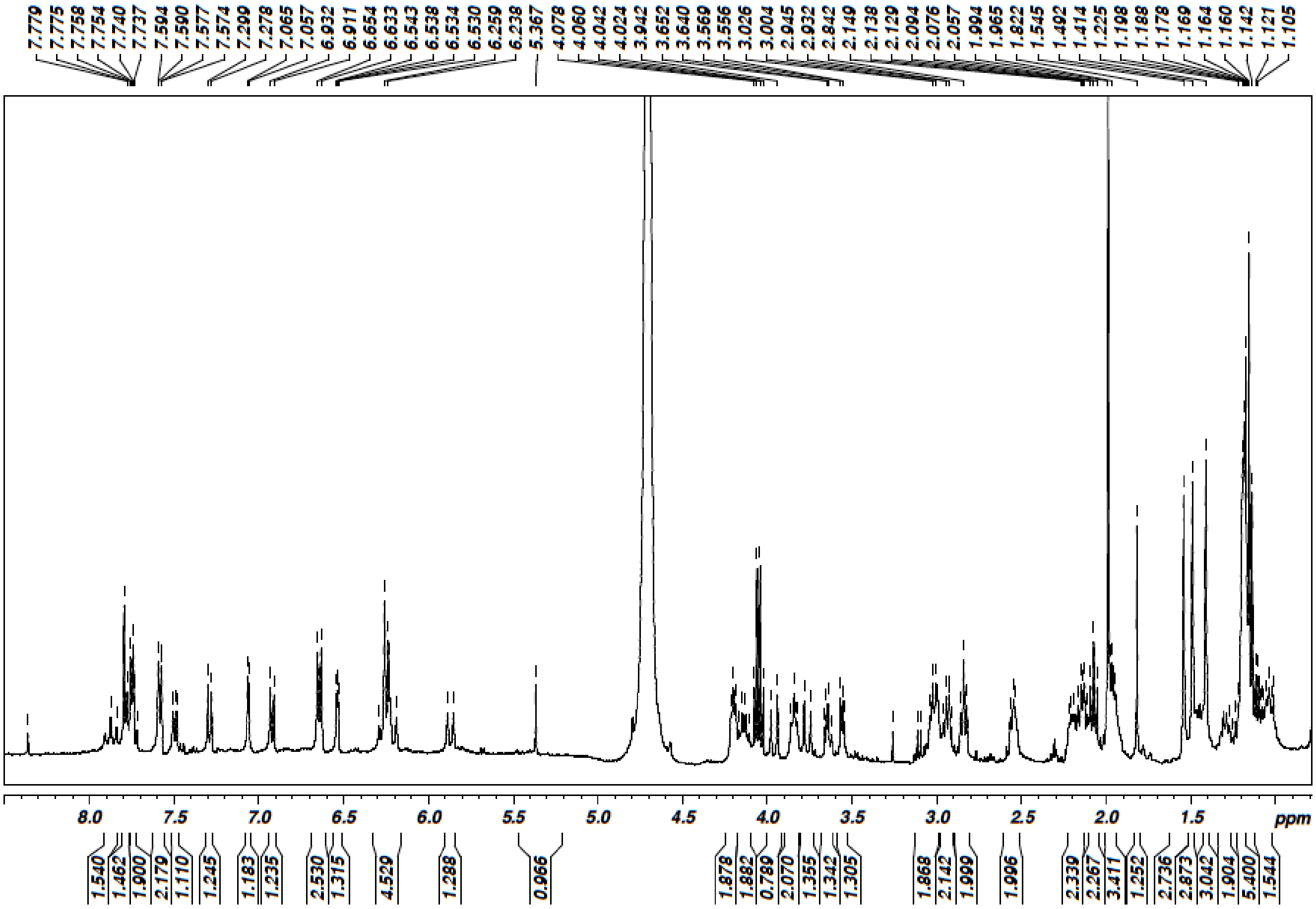

Data File D:\Chem32\1\Data\JINHA\New 2016-05-04 15-03-13\YJ-I-250000002.D
Sample Name: YJ-I-25

===

Acq. Operator : SYSTEM Seq. Line : 2
Acq. Instrument : HPLC-001 Location : 1
Injection Date : 5/4/2016 3:35:35 PM Inj : 1
Inj Volume : 10.000 µl

Different Inj Volume from Sample Entry! Actual Inj Volume : 5.000 µl
Sequence File : D:\Chem32\1\Data\JINHA\New 2016-05-04 15-03-13\New.S
Method : D:\Chem32\1\Data\JINHA\New 2016-05-04 15-03-13\ANAL_A20-60_C80-40_15MIN.M (Sequence Method)
Last changed : 5/4/2016 3:03:13 PM by SYSTEM

===

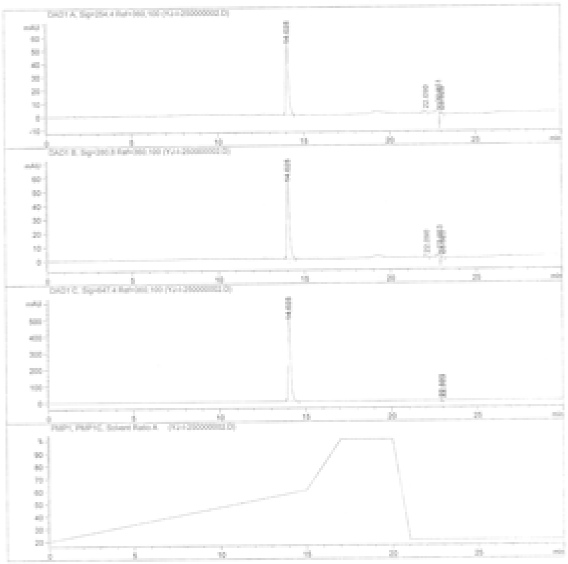

Data File D:\Chem32\1\Data\JINHA\New 2016-05-04 15-03-13\YJ-I-250000002.D
Sample Name: YJ-I-25

===

Acq. Operator : SYSTEM Seq. Line : 2
Acq. Instrument : HPLC-001 Location : 1
Injection Date : 5/4/2016 3:35:35 PM Inj : 1
Inj Volume : 10.000 µl

Different Inj Volume from Sample Entry! Actual Inj Volume : 5.000 µl
Sequence File : D:\Chem32\1\Data\JINHA\New 2016-05-04 15-03-13\New.S
Method : D:\Chem32\1\Data\JINHA\New 2016-05-04 15-03-13\ANAL_A20-60_C80-40_15MIN.M (Sequence Method)
Last changed : 5/4/2016 3:03:13 PM by SYSTEM

===

Peak #	RetTime [min]	Type	Width [min]	Area [mAU*s]	Height [mAU]	Area %
1	14.028	BB	0.1508	676.18964	67.53223	74.4960
2	22.090	BB	0.0996	9.63941	1.34534	1.0620
3	22.851	BV	0.1064	123.17556	14.60608	13.5703
4	22.929	VV	0.0604	35.29288	8.87608	3.8882
5	23.020	VB	0.1111	63.38794	7.45948	6.9835
Totals :				907.68542	99.81921	

Signal 2: DAD1 B, Sig=280,8 Ref=360,100

Peak #	RetTime [min]	Type	Width [min]	Area [mAU*s]	Height [mAU]	Area %
1	14.028	BB	0.1508	688.06165	68.71677	85.2914
2	22.090	BB	0.0951	7.60416	1.12209	0.9426
3	22.853	BV	0.0937	64.20507	8.73712	7.9588
4	22.927	VV	0.0506	17.22940	4.50268	2.1357
5	23.020	VB	0.1004	29.61807	3.90876	3.6714
Totals :				806.71834	86.98742	

Signal 3: DAD1 C, Sig=647,4 Ref=360,100

Peak #	RetTime [min]	Type	Width [min]	Area [mAU*s]	Height [mAU]	Area %
1	14.028	BB	0.1506	6023.11035	602.50580	99.0434
2	22.903	BV	0.0348	25.71258	10.43033	0.4228
3	22.983	VV	0.0751	32.46241	6.00627	0.5338
Totals :				6081.28535	618.94239	

10, MRS7395

Monoisotopic Mass, Even Electron Ions
442 formula(e) evaluated with 7 results within limits (up to 19 closest results for each mass)
Elements Used:
C: 0-100 H: 0-200 N: 11-11 O: 0-40 32S: 4-4
26-Jan-2017
xxx-26jan17-sample-neg 232 (4.291) Cn (Cen,5, 50.00, Ar); Sm (SG, 3x5.00); Sb (12,5.00)

TOF MS ES-
4.83e+002

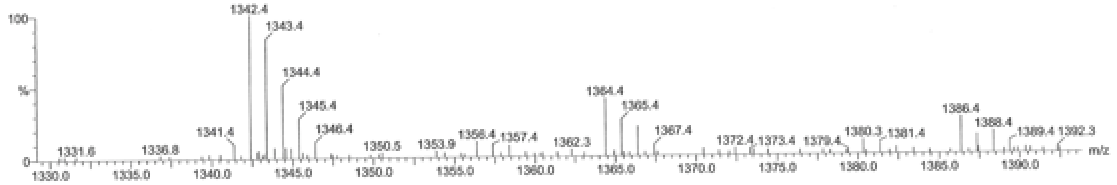

Minimum:				-2.0		
Maximum:		10.0	10.0	1000.0		
Mass	Calc. Mass	mDa	PPM	DBE	i-FIT	Formula
1342.4052	1342.4041	1.1	0.8	31.5	17.8	C61 H72 N11 O16 32S4
	1342.4065	-1.3	-1.0	0.5	73.1	C36 H84 N11 O34 32S4
	1342.4076	-2.4	-1.8	53.5	5.4	C79 H64 N11 O3 32S4
	1342.4006	4.6	3.4	9.5	52.7	C43 H80 N11 O29 32S4
	1342.4100	-4.8	-3.6	22.5	28.7	C54 H76 N11 O21 32S4
	1342.3983	6.9	5.1	40.5	10.5	C68 H68 N11 O11 32S4
	1342.4135	-8.3	-6.2	44.5	8.1	C72 H68 N11 O8 32S4

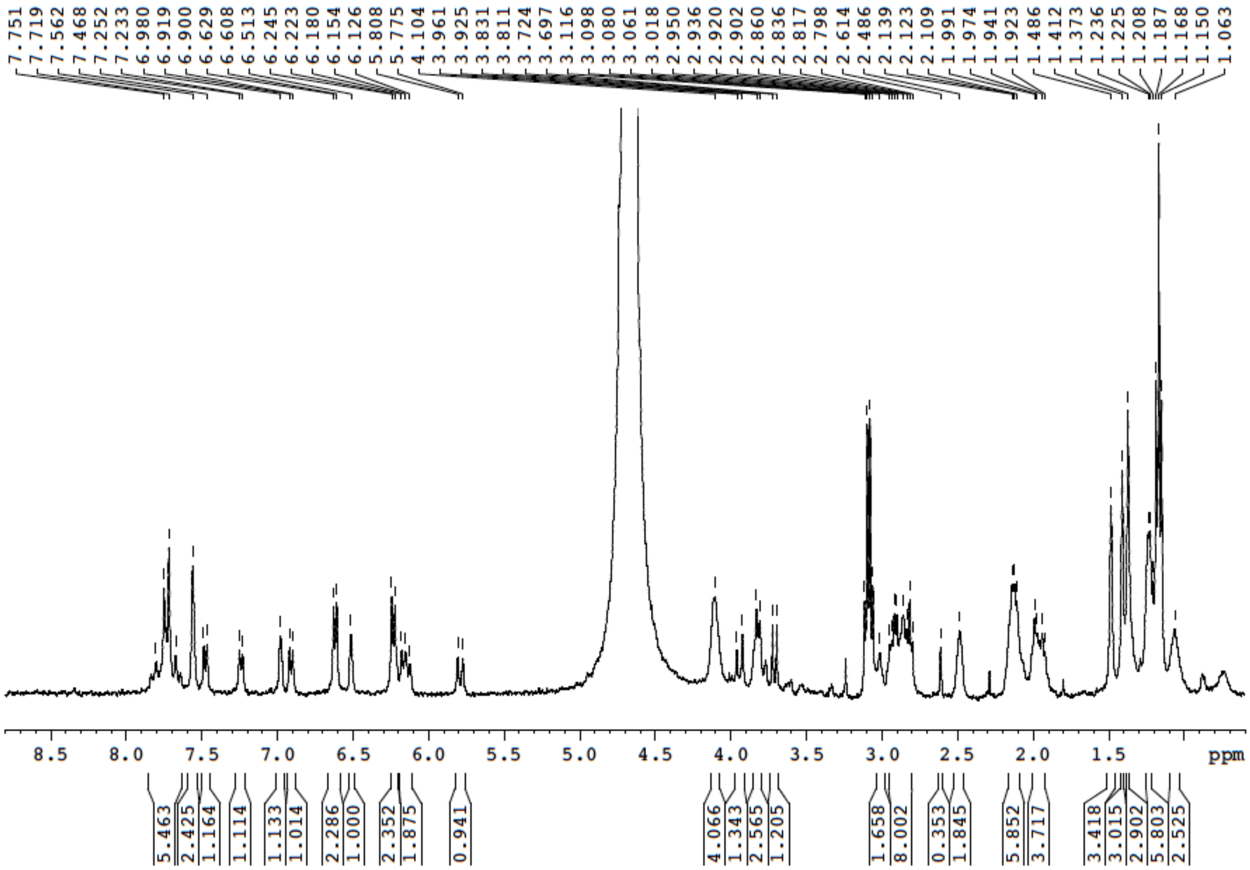

11, MRS7396

Monoisotopic Mass, Even Electron Ions
320 formula(e) evaluated with 5 results within limits (up to 19 closest results for each mass)
Elements Used:
C: 0-100 H: 0-200 N: 10-10 O: 0-40 F: 2-2 32S: 1-1 11B: 1-1

31-Jan-2017
rdx-31jan17-056 279 (5.159) Cn (Cen,5, 50.00, Ar); Sm (SG, 3x5.00); Sb (12,5.00)

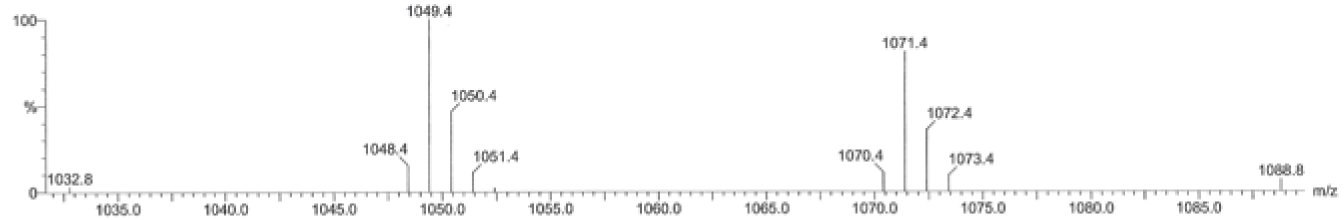

Minimum:				-2.0		
Maximum:		10.0	10.0	1000.0		
Mass	Calc. Mass	mDa	PPM	DBE	i-FIT	Formula
1049.4122	1049.4115	0.7	0.7	32.5	222.9	C55 H56 N10 O7 F2 32S 11B
	1049.4139	-1.7	-1.6	1.5	28.6	C30 H68 N10 O25 F2 32S 11B
	1049.4080	4.2	4.0	10.5	26.2	C37 H64 N10 O20 F2 32S 11B
	1049.4174	-5.2	-5.0	23.5	118.0	C48 H60 N10 O12 F2 32S 11B
	1049.4057	6.5	6.2	41.5	352.8	C62 H52 N10 O2 F2 32S 11B

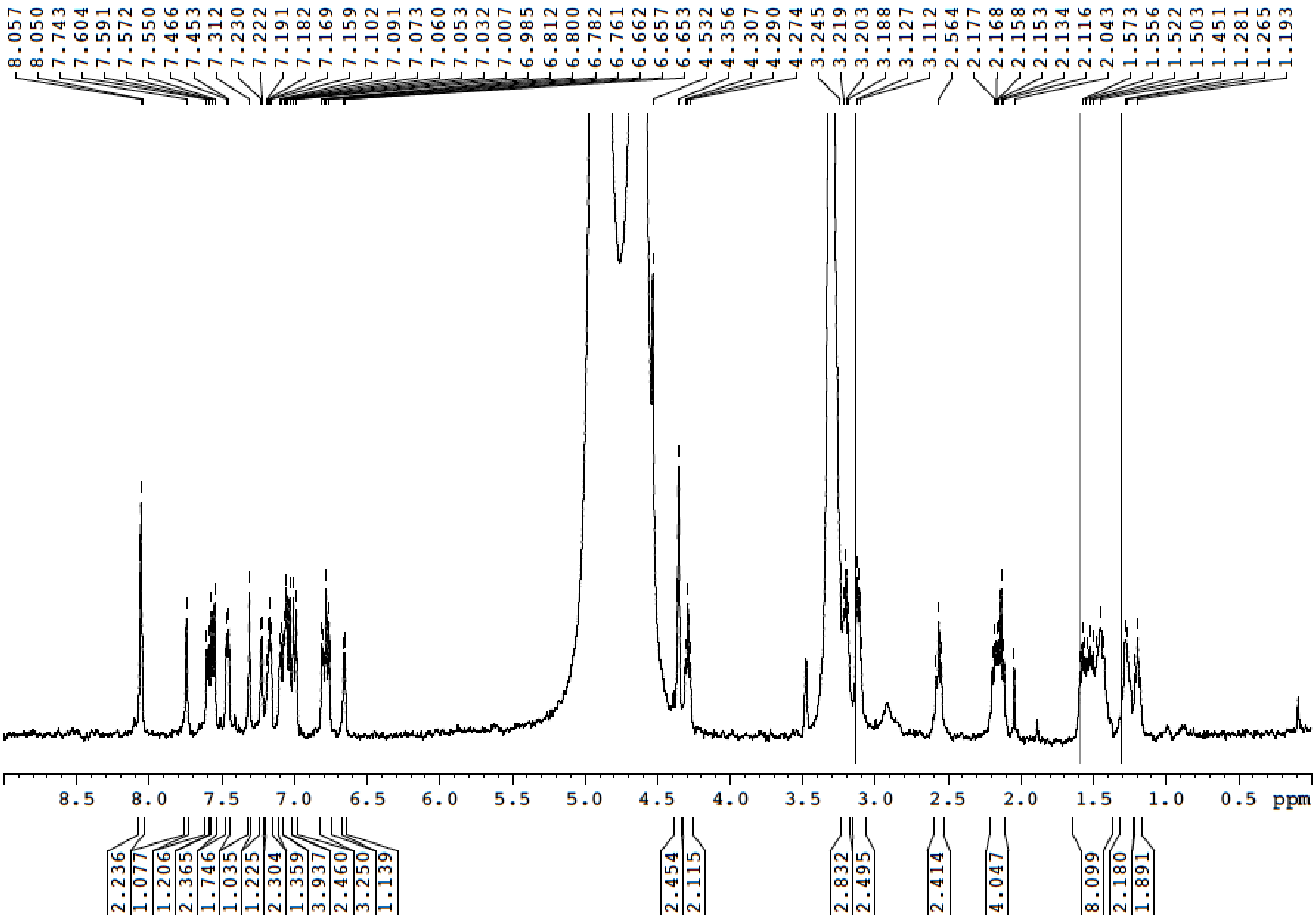

12, MRS7416

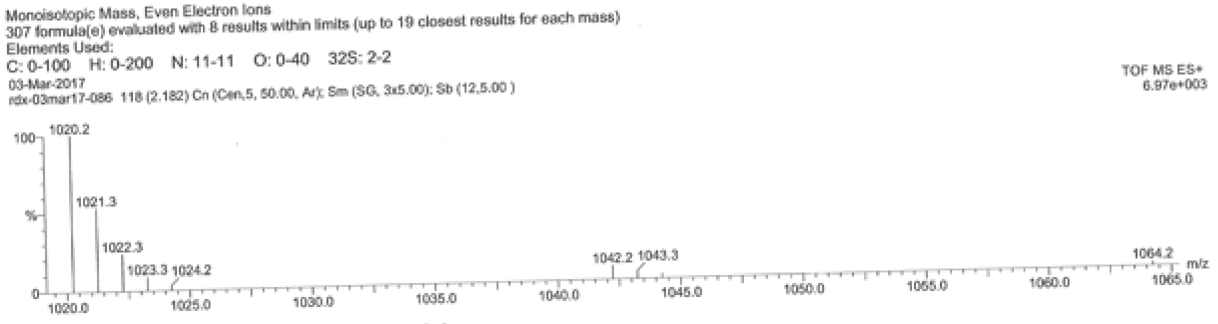

		mDa	PPM	DBE	i-FIT	Formula
Minimum:				-2.0		
Maximum:		10.0	10.0	1000.0		
Mass	Calc. Mass	mDa	PPM	DBE	i-FIT	Formula
1020.2411	1020.2405	0.6	0.6	31.5	50.1	C46 H42 N11 O13 32S2
	1020.2429	-1.8	-1.8	0.5	512.7	C21 H54 N11 O31 32S2
	1020.2440	-2.9	-2.8	53.5	167.5	C64 H34 N11 32S2
	1020.2370	4.1	4.0	9.5	292.5	C28 H50 N11 O26 32S2
	1020.2464	-5.3	-5.2	22.5	91.1	C39 H46 N11 O18 32S2
	1020.2346	6.5	6.4	40.5	65.5	C53 H38 N11 O8 32S2
	1020.2499	-8.8	-8.6	44.5	89.4	C57 H38 N11 O5 32S2
	1020.2311	10.0	9.8	18.5	150.6	C35 H46 N11 O21 32S2

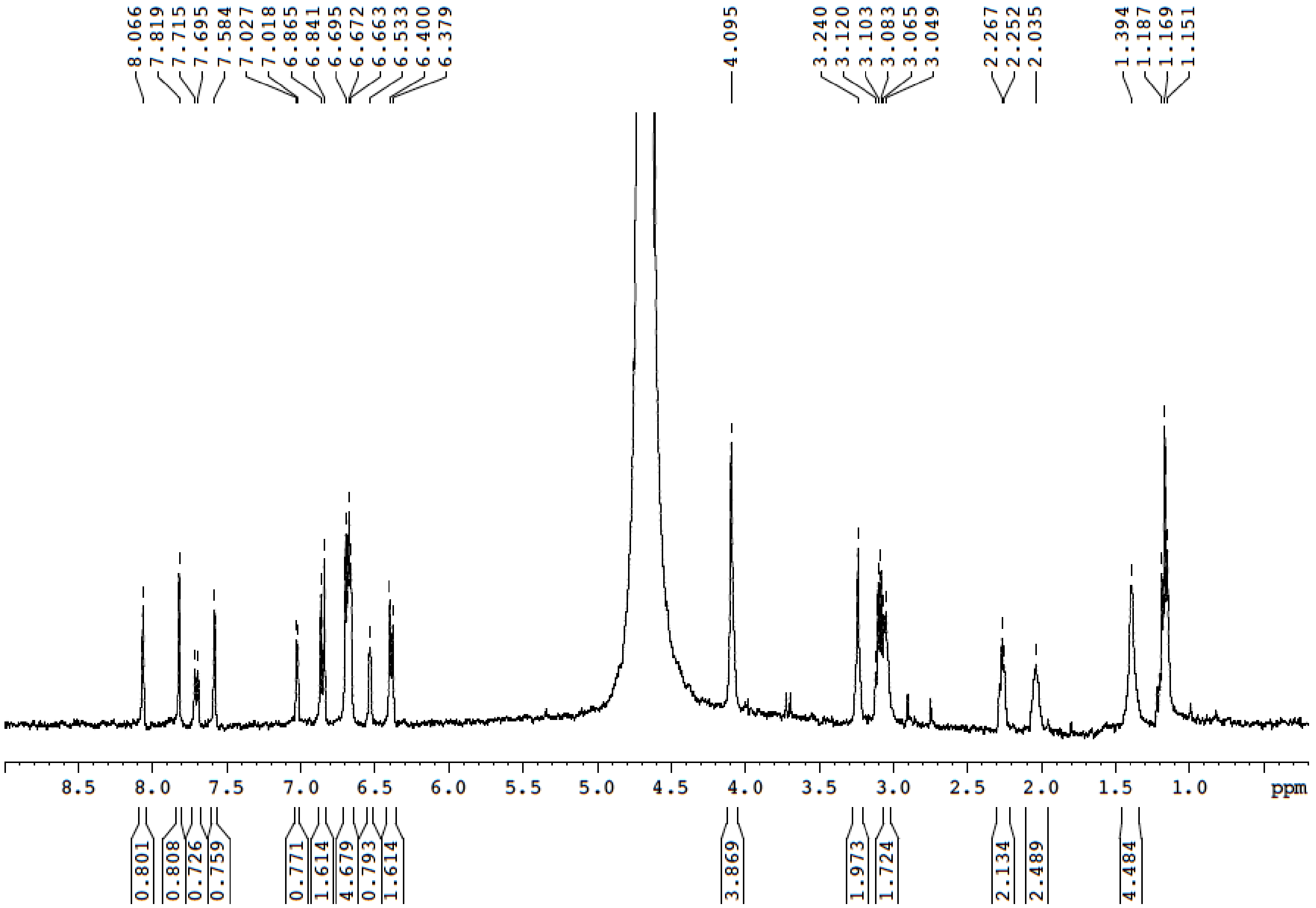

13, MRS7352

Monoisotopic Mass, Even Electron Ions
105 formula(e) evaluated with 4 results within limits (up to 19 closest results for each mass)
Elements Used:
C: 0-100 H: 0-200 N: 7-7 O: 0-40 32S: 1-1
12-Dec-2016
rdx-12dec16-012 183 (3.384) Cn (Cen,5, 50.00, Ar); Sm (SG, 1x3.00); Sb (12,5.00)

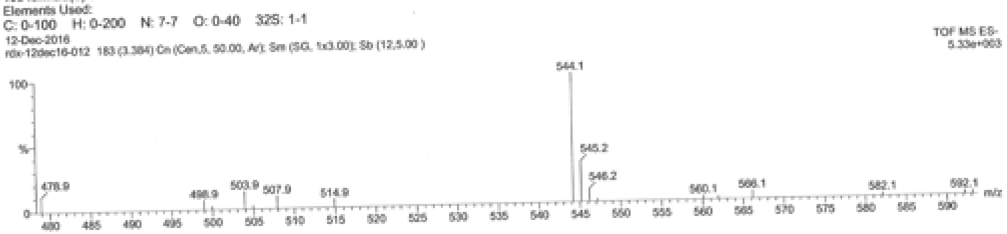

Minimum:				-2.0		
Maximum:		10.0	10.0	1000.0		
Mass	Calc. Mass	mDa	PPM	DBE	i-FIT	Formula
544.1401	544.1403	-0.2	-0.4	19.5	34.2	C26 H22 N7 O5 32S
	544.1344	5.7	10.5	28.5	59.5	C33 H18 N7 32S
	544.1462	-6.1	-11.2	10.5	84.3	C19 H26 N7 O10 32S
	544.1309	9.2	16.9	6.5	158.4	C15 H26 N7 O13 32S

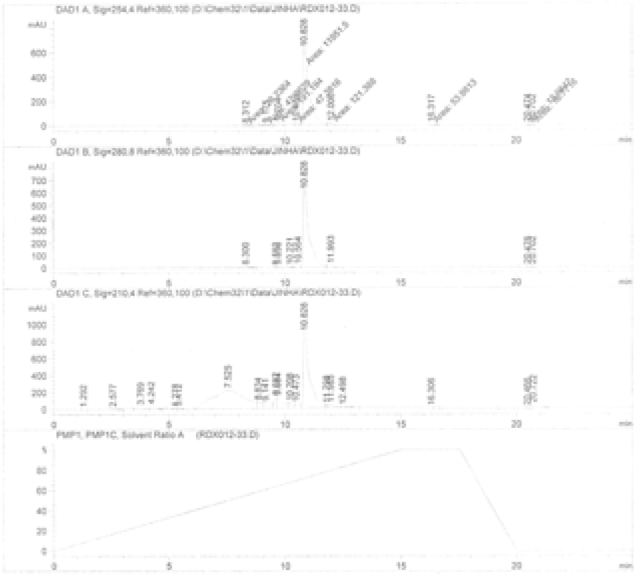

Signal 1: DAD1 A, Sig=254,4 Ref=360,100

Peak #	RetTime [min]	Type	Width [min]	Area [mAU*s]	Height [mAU]	Area %
1	8.312	MM	0.3210	26.23638	1.36222	0.2104
2	9.175	MM	0.3816	47.96289	2.09474	0.3846
3	9.704	MM	0.1867	191.19376	17.06560	1.5332
4	10.486	MM	0.1954	47.38156	4.04104	0.3800
5	10.828	MM	0.2401	1.19515e4	829.54095	95.8390
6	12.006	MM	0.1946	121.38776	10.39646	0.9734
7	16.317	MM	0.2314	53.98130	3.88733	0.4329
8	20.474	MM	0.0692	13.98472	3.36783	0.1121
9	20.702	MM	0.0850	16.77163	3.28925	0.1345
Totals :				1.24704e4	875.04543	

Signal 2: DAD1 B, Sig=280,8 Ref=360,100

Peak #	RetTime [min]	Type	Width [min]	Area [mAU*s]	Height [mAU]	Area %
1	8.300	BB	0.2367	68.20911	4.13051	0.5514
2	9.593	BV	0.0725	8.43298	1.80643	0.0682
3	9.696	VB	0.1355	26.96090	2.77155	0.2180
4	10.221	BV	0.1309	16.29919	1.84790	0.1318
5	10.564	VV	0.2031	100.01395	6.30378	0.8085
6	10.828	VB	0.1914	1.18983e4	809.86029	96.1875
7	11.993	BB	0.2353	228.84384	14.10428	1.8500
8	20.476	BB	0.0649	9.94817	2.27815	0.0804
9	20.702	BB	0.0868	12.89487	2.06762	0.1042
Totals :				1.23699e4	845.17053	

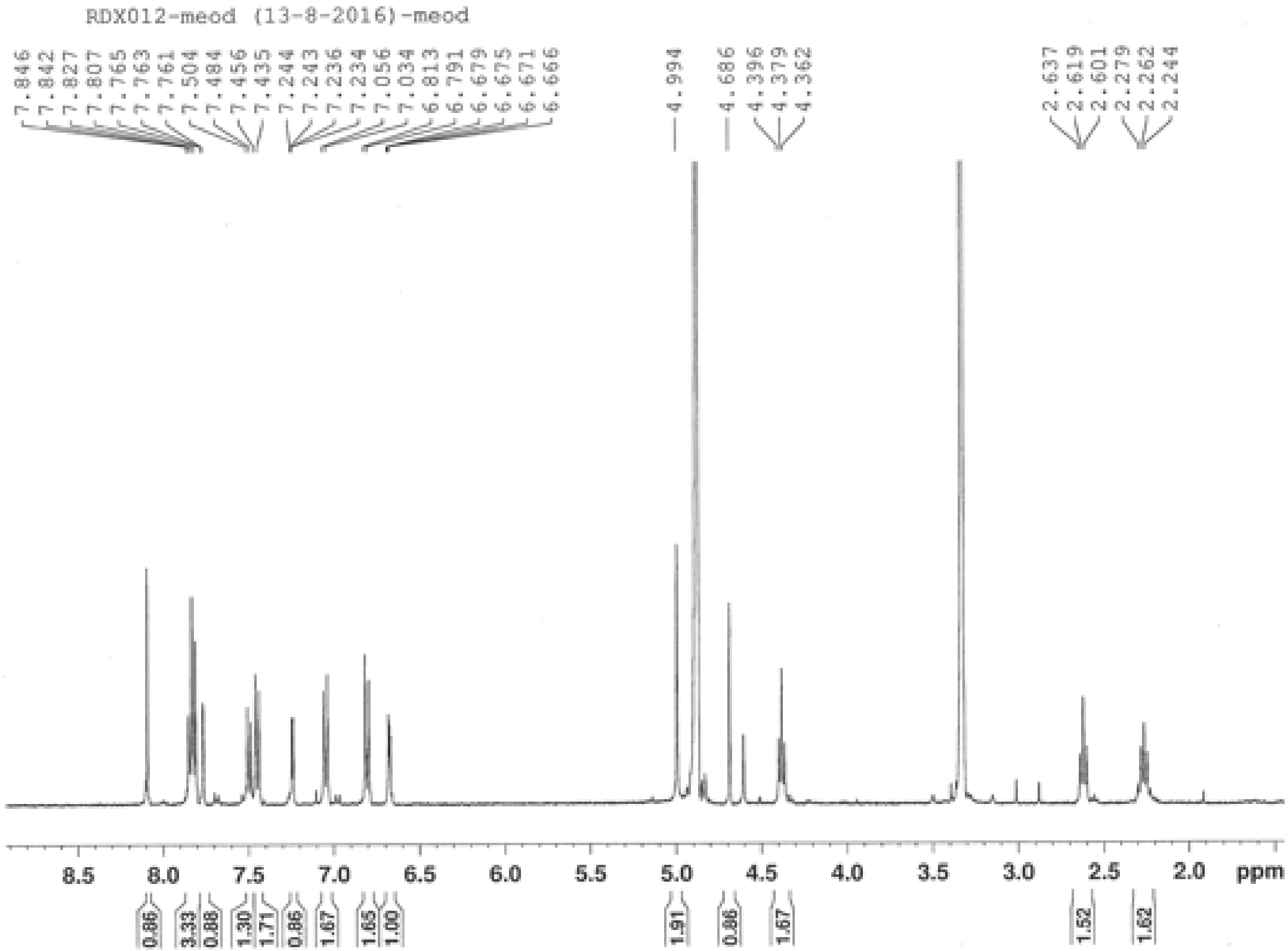
RDX012-meod (13-8-2016)-meod
7.846
7.842
7.827
7.807
7.765
7.763
7.761
7.504
7.484
7.456
7.435
7.244
7.243
7.236
7.234
7.056
7.034
6.813
6.791
6.679
6.675
6.671
6.666
4.994
4.686
4.396
4.379
4.362
2.637
2.619
2.601
2.279
2.262
2.244
8.5
8.0
7.5
7.0
6.5
6.0
5.5
5.0
4.5
4.0
3.5
3.0
2.5
2.0
ppm
0.86
3.33
0.88
1.30
1.71
0.86
1.67
1.65
1.00
1.91
0.86
1.67
1.52
1.62

UV absorption of MRS 7322

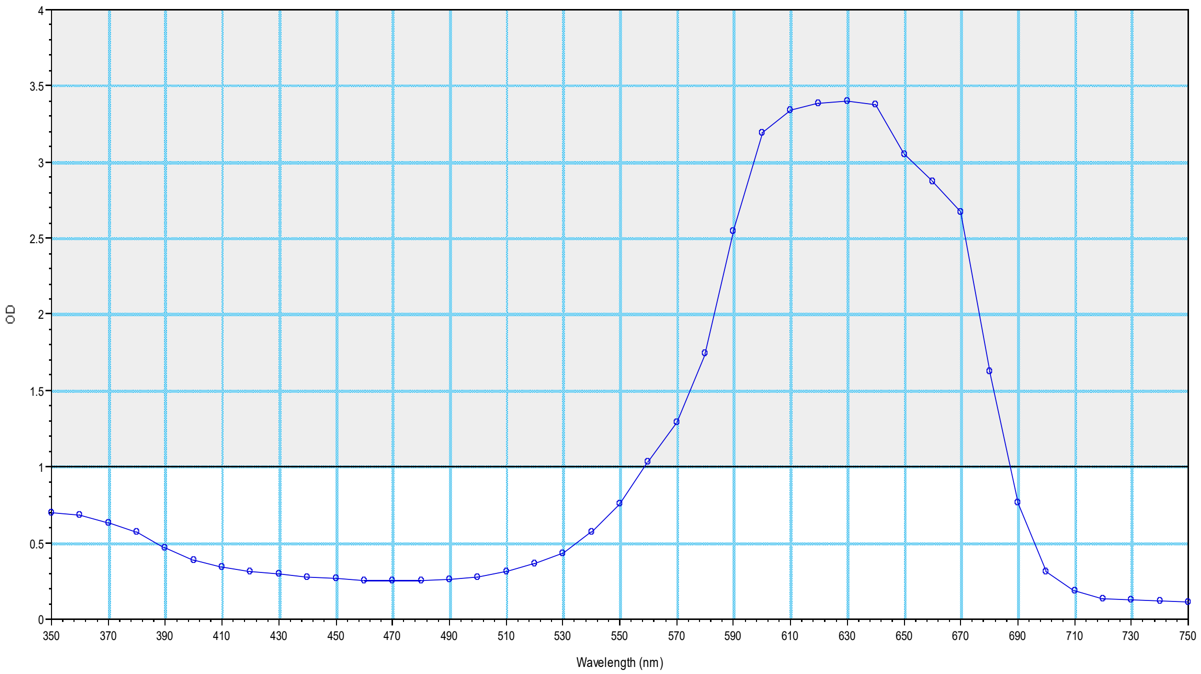

Lambda Maxium at 630 nm

Absorption: 3.4

Extinction Coefficient of Alexa 647: 270,000 $cm^{-1}M^{-1}$

Path Length: 1 cm

MW: 1382.45

Volume: 500 µL

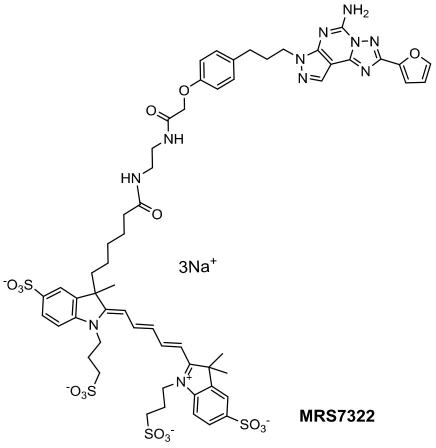

MRS7322

A = εbc

3.4 = 270000 x 1 x c

c = 3.4/270000 = 1.26 x 10^{-5} M

c = 12.6 µM

c = m/v

1.26 x 10^{-5} = m/5 x 10^{-4}

m = 6.3 x 10^{-9} mol

m = amount/M.W

6.3 x 10^{-9} M = amount/ 1382.45

amount = 8.71 µg
